# Supplementary material for: TGF-β Signaling Plays an Essential Role in the Lineage Specification of Mesenchymal Stem/Progenitor Cells in Fetal Bone Marrow
Source: Stem Cell Reports. 2019 Jun 13;13(1):48–60. doi: 10.1016/j.stemcr.2019.05.017 (PMC6626889; doi:10.1016/j.stemcr.2019.05.017)
Supplement: Document S1. Supplemental Experimental Procedures and Figures S1–S7 [file mmc1.pdf]

Stem Cell Reports, Volume *13*

## Supplemental Information

# TGF-β Signaling Plays an Essential Role in the Lineage Specification of Mesenchymal Stem/Progenitor Cells in Fetal Bone Marrow

**Grazia Abou-Ezzi, Teerawit Supakorndej, Jingzhu Zhang, Bryan Anthony, Joseph Krambs, Hamza Celik, Darja Karpova, Clarissa S. Craft, and Daniel C. Link**

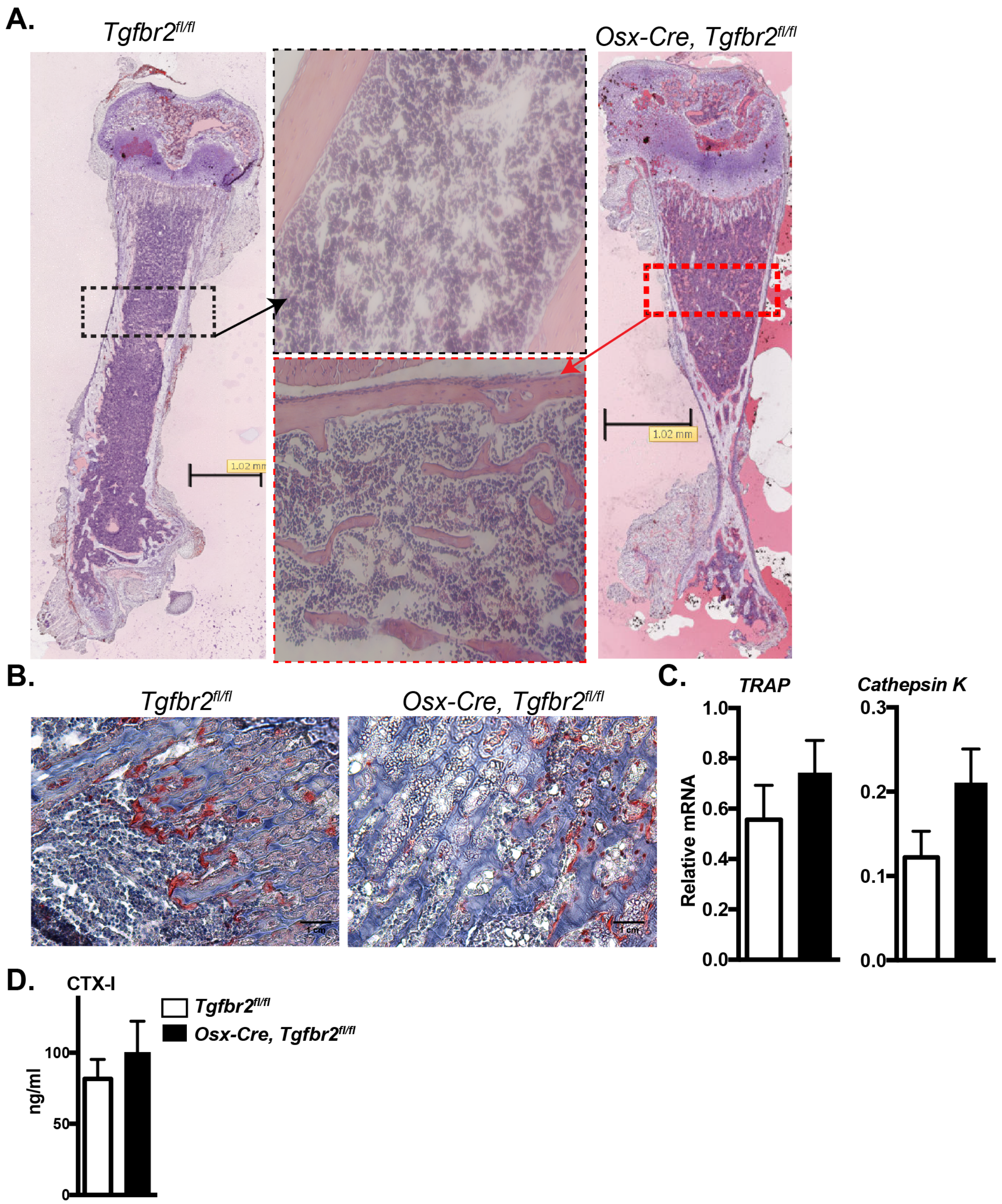

A.
Tgfbr2fl/fl
Osx-Cre, Tgfbr2fl/fl
1.02 mm
1.02 mm
B.
Tgfbr2fl/fl
Osx-Cre, Tgfbr2fl/fl
C.
TRAP
Cathepsin K
Relative mRNA
1.0
0.8
0.6
0.4
0.2
0.0
0.3
0.2
0.1
0.0
D.
CTX-I
ng/ml
100
50
0
Tgfbr2fl/fl
Osx-Cre, Tgfbr2fl/fl

A. CAR cells Perilipin

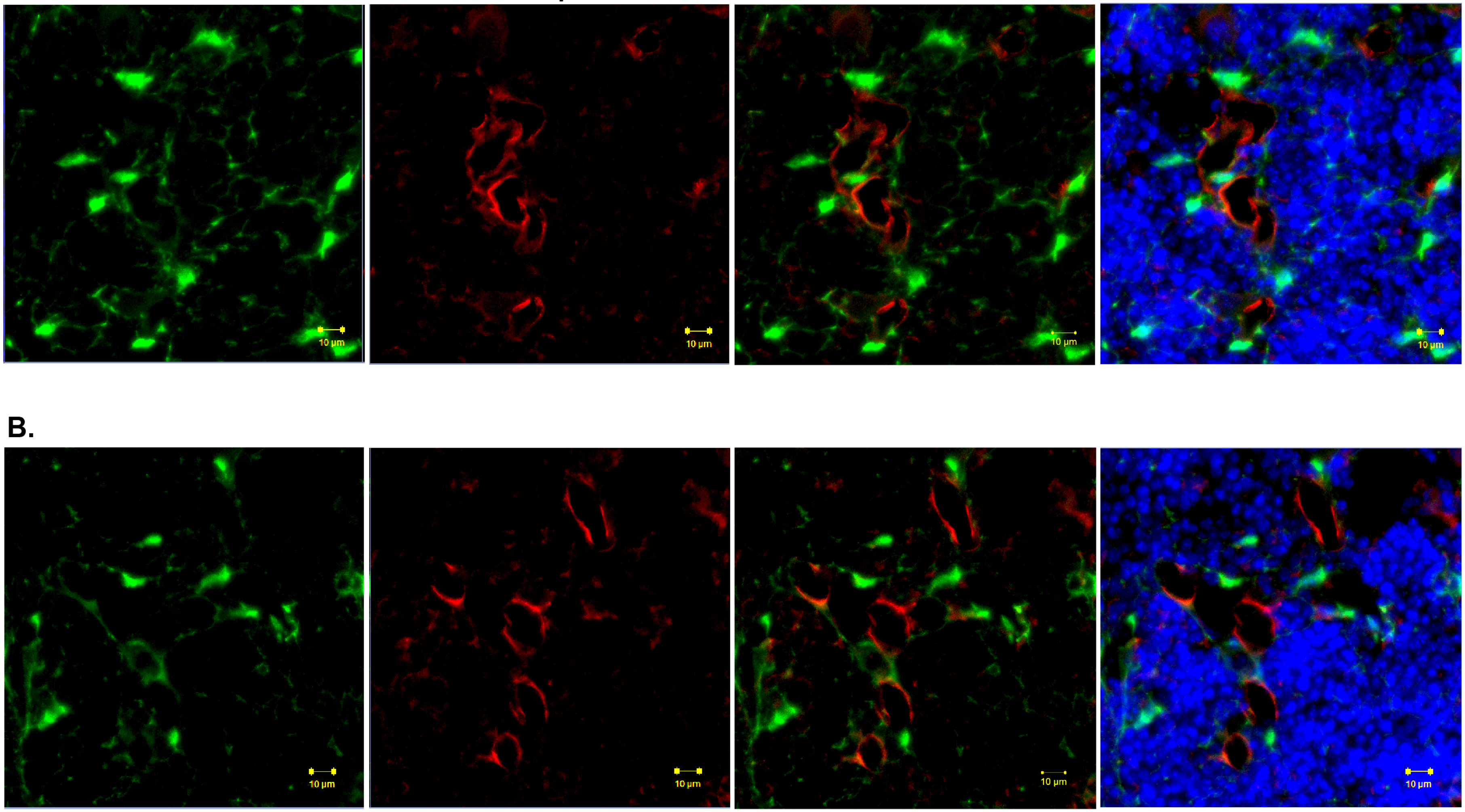


B.

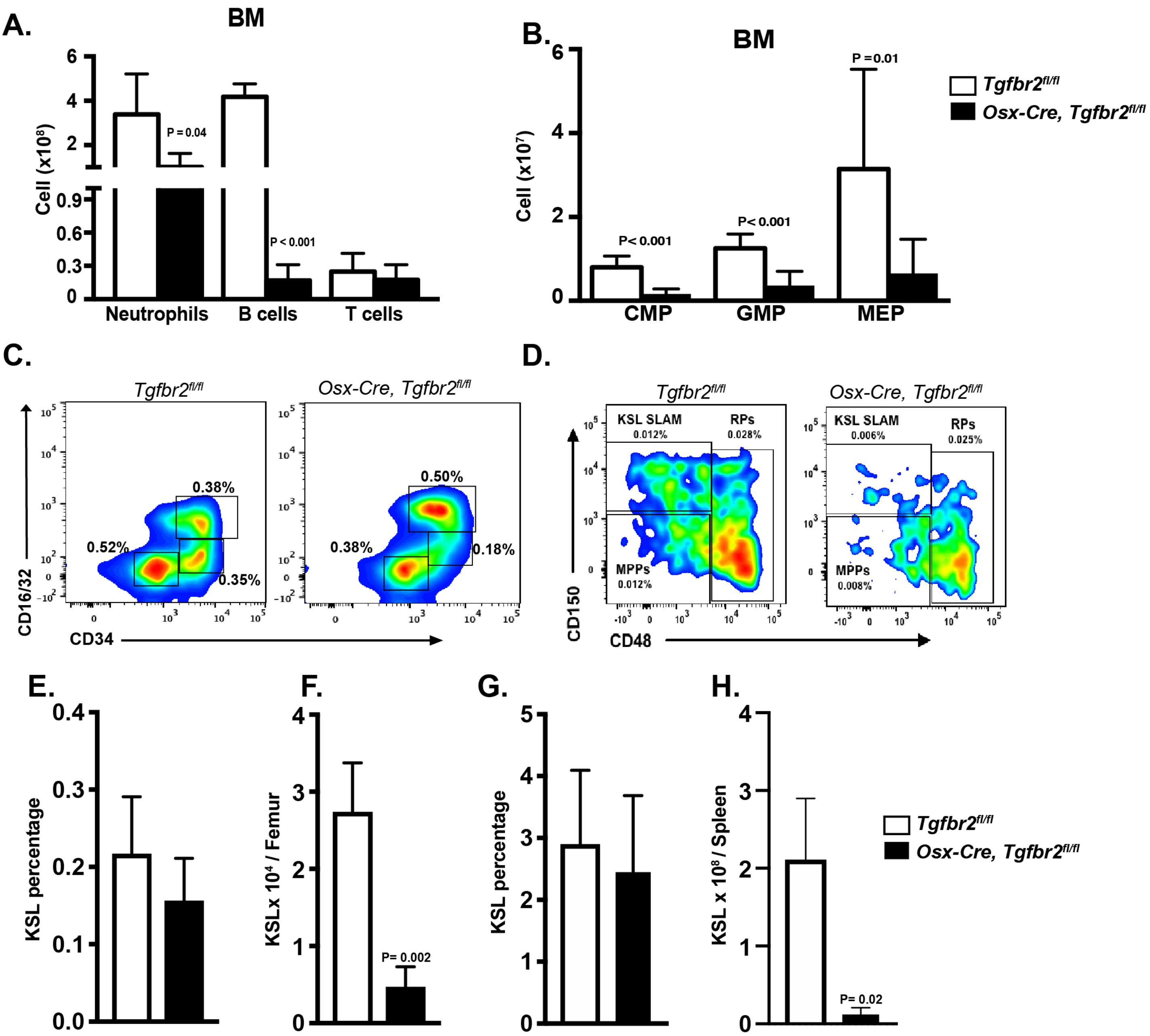
A.
BM
Cell (x10⁸)
P = 0.04
P < 0.001
Neutrophils
B cells
T cells
B.
BM
Cell (x10⁷)
P < 0.001
P < 0.001
P = 0.01
CMP
GMP
MEP
Tgfbr2fl/fl
Osx-Cre, Tgfbr2fl/fl
C.
Tgfbr2fl/fl
Osx-Cre, Tgfbr2fl/fl
0.38%
0.52%
0.35%
0.50%
0.38%
0.18%
CD16/32
CD34
D.
Tgfbr2fl/fl
Osx-Cre, Tgfbr2fl/fl
KSL SLAM
0.012%
RPs
0.028%
MPPs
0.012%
KSL SLAM
0.006%
RPs
0.025%
MPPs
0.008%
CD150
CD48
E.
KSL percentage
F.
KSLx 10⁴ / Femur
P= 0.002
G.
KSL percentage
H.
KSL x 10⁸ / Spleen
P= 0.02
Tgfbr2fl/fl
Osx-Cre, Tgfbr2fl/fl

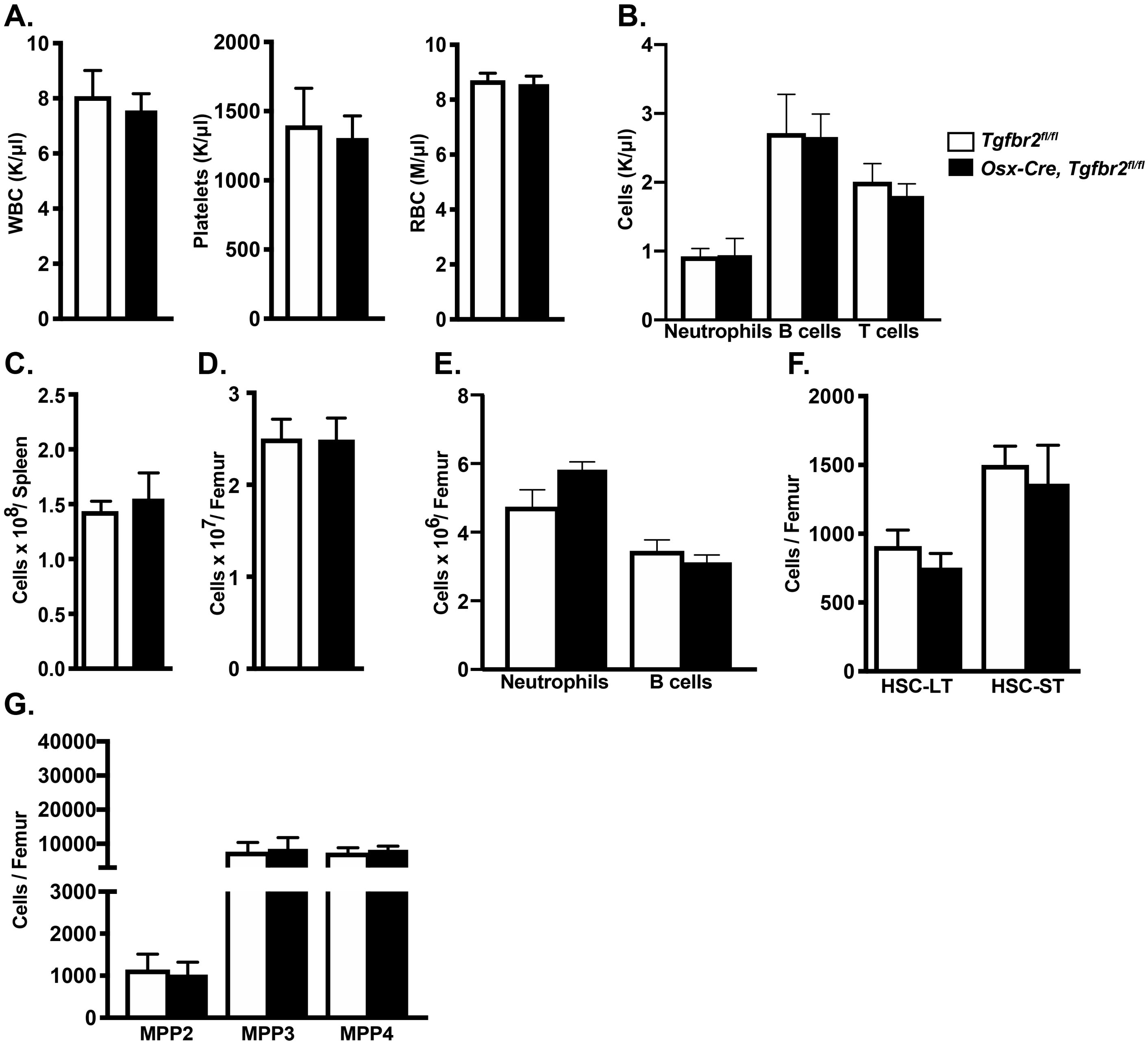
A.
WBC (K/µl)
Platelets (K/µl)
RBC (M/µl)
B.
Cells (K/µl)
Neutrophils B cells T cells
Tgfbr2fl/fl
Osx-Cre, Tgfbr2fl/fl
C.
Cells x 10^8/ Spleen
D.
Cells x 10^7/ Femur
E.
Cells x 10^6/ Femur
Neutrophils
B cells
F.
Cells / Femur
HSC-LT
HSC-ST
G.
Cells / Femur
MPP2
MPP3
MPP4

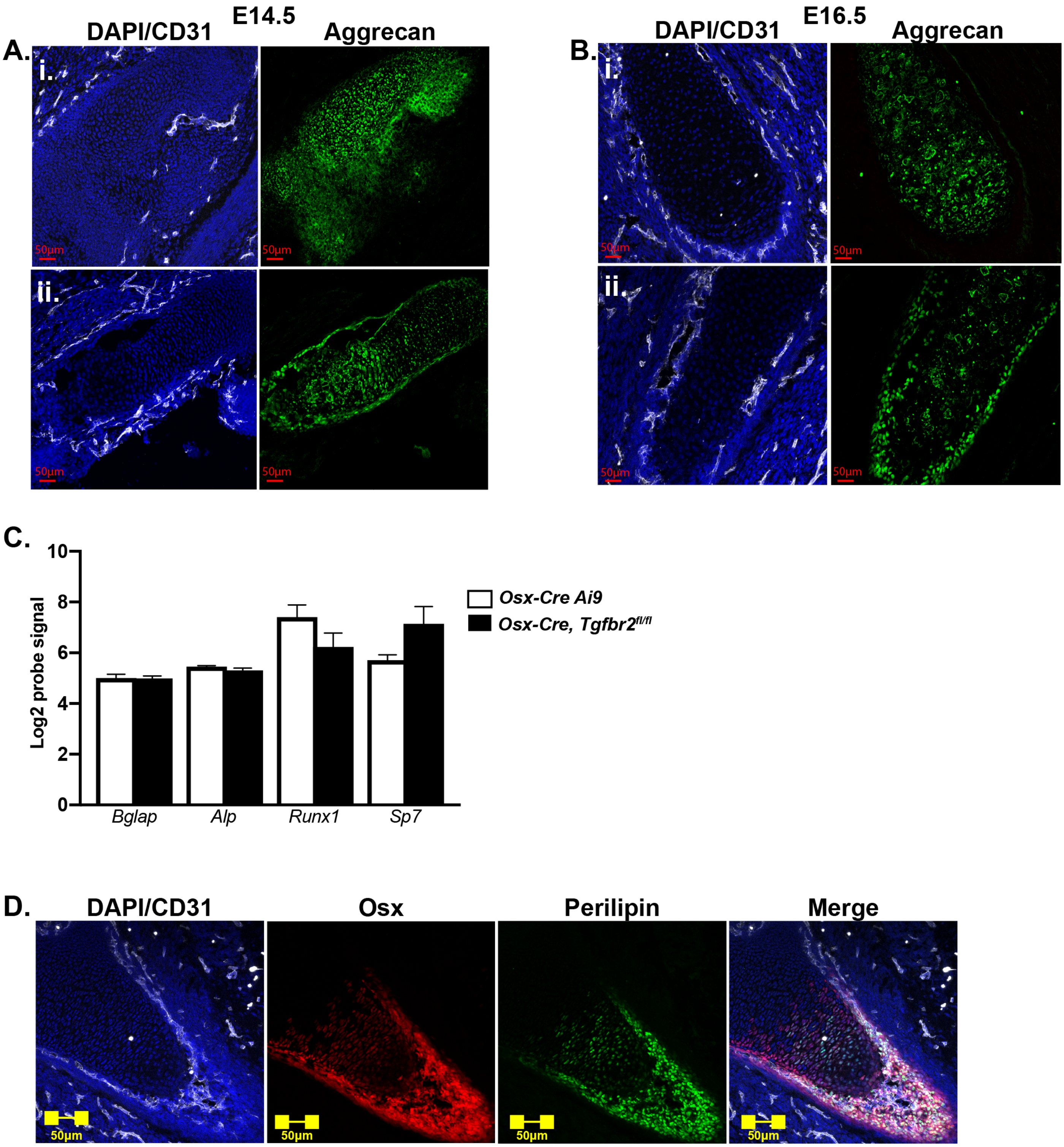

E14.5
E16.5
A.
B.
DAPI/CD31
Aggrecan
i.
ii.
50µm
C.
Log2 probe signal
Osx-Cre Ai9
Osx-Cre, Tgfbr2fl/fl
Bglap
Alp
Runx1
Sp7
D.
DAPI/CD31
Osx
Perilipin
Merge
50µm

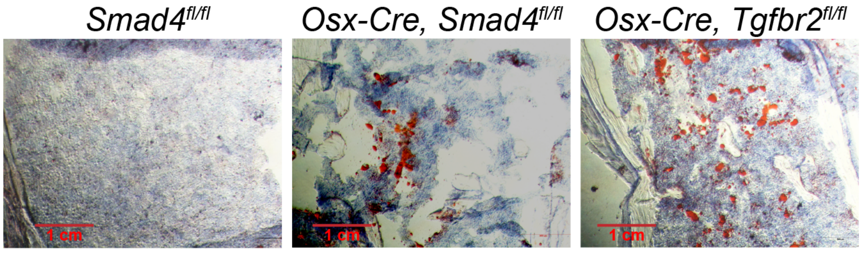
*Smad4*$^{fl/fl}$
*Osx-Cre, Smad4*$^{fl/fl}$
*Osx-Cre, Tgfbr2*$^{fl/fl}$
1 cm
1 cm
1 cm

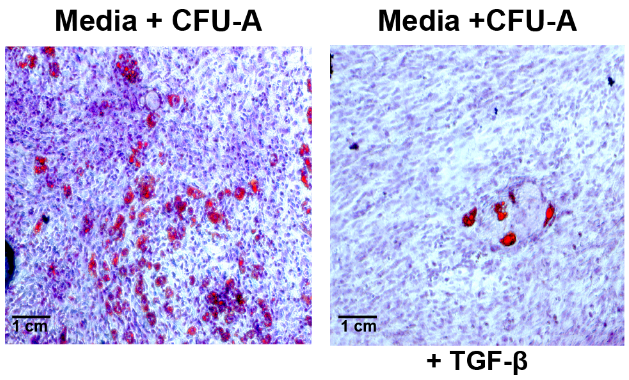
Media + CFU-A
Media +CFU-A
1 cm
1 cm
+ TGF-β

## SUPPLEMENTAL FIGURE LEGENDS

**Figure S1**. **Related to Figure 1. Loss of TGF-β signaling in mesenchymal cells does not alter osteoclasts. A.** Representative photomicrographs of H&E stained femur sections from *Tgfbr2$^{fl/fl}$* and *Osx-CreTgfbr2$^{fl/fl}$* mice. **B.** Representative photomicrographs of femur sections stained with tartrate-resistant acidic phosphatase (TRAP, red). Original magnification 20X. **C.** RNA expression of the indicated osteoclast marker gene relative to β-actin mRNA is shown (n=5)
Osteoclasts functional genes marker of total bone marrow mRNA expression relative to β-actin. **D.** Serum C-terminal telopeptide of type I collagen (CTX-1) (n = 11-12). Data represent the mean ± SEM.

**Figure S2. Related to Figure 3. CAR cells are distinct from perilipin$^+$ adipocytes. (A, B)** Representative images of femur sections from Tgfbr2$^{fl/fl}$ *Cxcl12$^{gfp}$* mice showing CXCL12-GFP (green), perilipin (red), and DAPI (blue).

**Figure S3**. **Related to Figure 4. Loss of TGF-β signaling in mesenchymal cells results in perturbed hematopoiesis. A-B.** The absolute number of the indicated cell type per pelvis and combined lower limbs is shown (n = 5). CMP, (common myeloid progenitor), GMP (granulocytes-macrophages progenitors), and MEP (myeloid-erythrocytes progenitors). **C**. Representative dot plots showing the gating strategy to identify CMP, GMP, and MEP; data are gated on c-Kit$^+$, lineage$^-$, Sca1$^-$ cells. **D**. Representative dot plots showing the gating strategy to identify HSCs; data are gated on c-Kit$^+$, lineage$^-$, Sca1$^+$ cells. RP, restricted progenitors, MPP, multipotent progenitors. **E-F.** Percentage and absolute number of bone marrow KSL cells. **G-H.** Percentage and absolute number of spleen KSL cells.

**Figure S4. Related to Figure 5. Post-natal deletion of *Tgfbr2* in mesenchymal cells is not required for basal hematopoiesis. A.** Peripheral blood counts. WBC, white blood cells; and RBC, red blood cells (n=6). **B.** Number of neutrophils, B cells, and T cells in the blood is shown (n=6). **C-D**. Spleen and bone marrow cellularity (per femur) (n=6). **E.** Number of neutrophils and B cells per femur. **F.** Number of HSC-LT (Lin$^-$ kit$^+$ Sca1$^+$ CD48$^-$ CD150$^+$ Flt3$^-$) and HSC-ST (Lin$^-$ kit$^+$ Sca1$^+$ CD48$^-$ CD150$^-$ Flt3$^-$) per femur. **G.** Number of MMP2 (Lin$^-$ kit$^+$ Sca1$^+$ CD48$^+$ CD150$^+$ Flt3$^-$), MMP3 (Lin$^-$ Ckit$^+$ Sca1$^+$ CD48$^+$ CD150$^-$ Flt3$^-$), and MPP4 (Lin$^-$ kit$^+$ Sca1$^+$ CD48$^+$ CD150$^-$ Flt3$^+$).

**Figure S5**. **Related to Figure 6. TGF-β signaling in *Osx-Cre* targeted mesenchymal progenitors is not required for chondrocyte development. A-B.** Representative images E14.5 (**A**) or E16.5 (**B**) hindlimb sections from *Tgfbr2$^{fl/fl}$* (i) or *Osx-Cre, Tgfbr2$^{fl/fl}$* (ii) mice showing aggrecan (green, chondrocytes), CD31 (white, vasculature) and DAPI (blue, nuclei) staining. **C.** RNA expression profiling was performed on sorted TdTomato+ lineage- cells isolated from the hindlimb of E16.5 Osx-Cre Ai9 and Osx-*Cre, Tgfbr2$^{fl/fl}$ Ai9* mice. Shown are log2 probe signals for *Bglap, Alp, Runx1 and Sp7*. **D.** *Osterix-Cre* targeted mesenchymal cells express perilipin. Representative images of E16.5 fetal hindlimb sections from Osx-*Cre, Tgfbr2$^{fl/fl}$ Ai9* mice showing tdTomato (red, *Osx-Cre* targeted cells), CD31 (white, vasculature), perilipin (green, adipocytes cells) and DAPI (blue, nuclei) staining. Original magnitude 20X.

**Figure S6**. **Related to figure 7. Loss of *Smad4* in *Osx-Cre* targeted mesenchymal cells results in altered adipogenesis.** Representative images of femur sections showing oil red staining. Original magnification, 20X.

**Figure S7**. **Related to figure 7. TGF-β suppresses adipogenesis of cultured fetal mesenchymal progenitors.** Mesenchymal progenitors were isolated from wildtype E16.5 hindlimbs and cultured for 7 days in the (media) presence of dexamethasone, insulin and indomethacin to induce adipocyte differentiation (CFU-A). Where indicated, TGF-β was included in the culture. Shown are representative images of cultures stained for oil red to show adipocytes.

**Table S1. Related to figure 6. Differentially expressed genes in *Osx-Cre* targeted (tdTomato+) cells from E16.5 hindlimbs.**

### Experimental Procedure

### Contact for Reagents and Resource Sharing

Further information and requests for resources and reagents should be directed to and will be fulfilled by the Lead Contact, Daniel C. Link (danielclink@wustl.edu).

**Experimental Model and Subject Details**

Mice and Animal Housing

*Osx1-GFP::Cre (Rodda and McMahon (2006),* Ai9 (Madisen, Zwingman et al. 2010), *TβRII* $^{fl/fl}$ (Leveen 2002), *Dmp1-cre* (Lu, Xie et al. 2007), and *Smad4* $^{fl/fl}$ (*Smad4*$^{tm2.1Cxd}$/J) mice were obtained from The Jackson Laboratory. *Cxcl12*$^{gfp}$ mice (Nagasawa, Hirota et al. 1996) were a gift from Dr.Takashi Nagasawa (Kyoto University, Japan). Mice were crossed in-house to generate *Osx-Cre Tgfbr2*$^{flfl}$*, Osx-Cre Tgfbr2*$^{flfl/Ai9}$*, Osx-Cre Tgfbr2*$^{flfl}$*Cxcl12*$^{gfp}$*, Osx-Cre*$^{Ai9}$*, Dmp-1-Cre Tgfbr2*$^{flfl}$*, and Osx-Cre Smad4*$^{flfl}$ Ai9 mice on a C57Bl/6 background. To suppress the *Osx-Cre* transgene, mice were maintained on doxycycline chow (200 mg/serving). All experiments were done using 3 weeks old mice unless stated otherwise. An equal number of male and female mice were used. Mice were maintained under standard pathogen-free conditions according to methods approved by the Washington University Animal Studies Committee.

Flow cytometry

Peripheral blood, bone marrow, and spleen mononuclear cell preparations were lysed in Tris-buffered ammonium chloride (pH 7.2) buffer for 5 minutes at 4°C. Cells were then incubated with the indicated antibody at 4°C for 30 minutes in phosphate buffered saline (PBS) containing 1mM ethylenediaminetetraacetic acid (EDTA) and 0.2% (weight/volume) bovine serum albumin (BSA). The HSPC panel included Pe-Cy7-conjugated CD117 (2B8); BV711-conjugated Ly-6A/E; BV605-conjugated CD150 (TC15-12F12.2) BV421-conjugated CD48 (HM48-1); APC-conjugated CD16/32 (2.4G2); FITC-conjugated CD34 (RAM34); and PE-conjugated CD135 (A2F10.1) and the following APC-Cy7-conjugated antibodies: CD3e (145-2C11), B220 (RA3-6B2), Gr1 (RB6-8C5), Ter119 (TER-119), and CD11b (M1/70). The CAR cell panel included the following BV421-conjugated antibodies against CD45 (30-F11), B220, Gr1, and CD11b. Cells were acquired using a FACSAria III flow cytometer (BD biosciences, San Jose, CA, USA) and analyzed using FlowJo software.

Cell sorting

Hindlimbs and forelimbs from embryonic day E16.5 were homogenized in PBS using a mortar and pestle. The cell suspension was then incubated in PBS containing 1.7 mg/ml collagenase type 1 (#17100017, ThermoFisher, Waltham, MA, USA), 1.7 mg/ml collagenase type 2 (#LS004174, Worthington, Lakewood, NJ, USA), and 1.7 mg/ml collagenase type 4 (#LS004188, Worthington) at 37°C while shaking for 10 minutes. The resulting cell suspension was filtered through CellTrics 50µm filters (Sysmex, Goerlitz, Germany) to remove cell clumps and then incubated at 4°C for 30 minutes in PBS containing 1mM EDTA and 0.2% BSA with the following panel of FITC-conjugated lineage antibodies: CD45, Gr1, CD11b, and B220. Tdtomato$^{+}$ lineage$^{-}$ cells were sorted using a Sony iCyt Synergy SY3200 (Synergy)" cell sorter (Sony, San Jose, CA, USA). CXCL12-GFP-bright CD45$^{-}$ lineage$^{-}$ cells were sorted from *Osx-Cre Tgfbr2*$^{flfl}$*Cxcl12*$^{gfp}$ *or Tgfbr2*$^{flfl}$*Cxcl12*$^{gfp}$ mice using a similar strategy.

Micro-computed tomography and osmium staining

Hindlimbs were incubated overnight at 4°C in 10% neutral buffered formalin and then embedded in 2% agarose. Femurs were scanned at 10µm voxel resolution, using a Scanco µCT 40 (Scanco Medical, Wayne, PA) calibrated using a hydroxyapatite phantom. Measurements of both cancellous and cortical bones were made based on reported guidelines (Bouxsein, Boyd et al. 2010). For cancellous bone, 100 slices (1mm) proximal to the growth plate (GP) were contoured to exclude the cortical bone, allowing cancellous bone volume/tissue volume (BV/TV) and bone mineral density (BMD) to be determined. For cortical bone, 20 slices (200 µm) located mid-diaphysis were contoured to exclude the marrow space and analyzed to determine cortical tissue mineral density (TMD) and cortical bone thickness. A threshold of 260 for cortical bone (on a 0–1000 scale) was maintained. For cancellous bone, a threshold of 175 was used for µCT. An additional analysis of cancellous bone was performed within the cortical bone region of interest after discovering the presence of cancellous bone mid-diaphysis in the mutant, but not control bones. Like cancellous bone at the GP, femurs were contoured to exclude the cortical bone and a threshold of 175 was maintained.

For osmium staining, hindlimbs were fixed with 10% neutral-buffer formalin, washed with water and decalcified in 14% EDTA, Ph 7.4, for 2 weeks. After washing again with water, 600µl Sorensen's phosphate buffer (pH 7.4) was added and then the hindlimbs incubated in a fume hood with 1% osmium tetroxide for 48 hours at room temperature. Hindlimbs were washed three times by incubating in 1 ml of Sorensen's buffer for 3 hours at room temperature with the last wash including an overnight incubation at room temperature.

Immunostaining of bone sections

Mouse hindlimbs were fixed in PBS containing 4% paraformaldehyde, pH 7.4, for 24 hours at 4°C. Bones were then decalcified in PBS containing 14% EDTA, pH 7.4, for 7 days at 4°C. Following incubation in PBS containing 30%

sucrose for 24 hours at 4°C, bones were embedded in optimal cutting temperature compound (OCT) (Sakura Finetek, Torrance, CA, USA). The tissue blocks were cut into 12µm sections using a Leica Cryo-Jane system (Leica Biosystems, Wetzlar, Germany). For immunostaining, the slides were first incubated in 0.1M Tris-Cl pH 7.5, 150mM NaCl, and 0.1% Tween 20 (TNT) buffer containing 10% donkey serum for 1 hour at room temperature. Sections were then incubated for 15 minutes at room temperature using the Avidin/Biotin Blocking Kit (SP-2001; Vector Laboratories, Burlingame, CA, USA). Sections were incubated with the primary antibody overnight at 4°C and, where applicable, then incubated with the secondary antibody at a 1 to 100 dilution for 1 hour at room temperature. The following antibodies were used: rabbit anti-perilipin at a 1:800 dilution (MilliporeSigma), anti-PECAM-1 at a 1:100 dilution (clone 2H8, MilliporeSigma), rabbit anti-aggrecan at a 1:100 dilution (AB1031, MilliporeSigma), and rabbit anti-osteocalcin at a 1:50 dilution (ab10911, ABCAM, Cambridge, United Kingdom). Finally, slides were mounted with ProLong Gold antifade reagent with DAPI (Life Technologies, Inc., Grand Island, NY, USA). Images were acquired using an LSM 700 confocal microscope (Carl Zeiss Microscopy, Peabody, MA, USA) and processed using Volocity software (PerkinElmer, Waltham, MA, USA).

Hematoxylin and eosin (H&E) staining of bone sections was performed using the Hematoxylin and Eosin Stain Kit (Vector Laboratories Burlingame, Ca. USA, Cat# H-3502). Oil red staining was performed using the Sigma Oil Red O kit per manufacturer's recommendations (MilliporeSigma). Sections were mounted with Organo/Limonene Mount™ (MilliporeSigma), and images were acquired using an Hamamatsu Nanozoomer (Hamamatsu Photonics, Hamamatsu City, Japan).

Quantitative reverse-transcription PCR

Total bone marrow RNA was obtained by flushing femurs with 1 ml of Trizol (Invitrogen). RNA was prepared according to the manufacturer's specification. One-step quantitative reverse-transcription PCR was performed using the TaqMan Universal PCR Master Mix (Applied Biosystems) using no template and no reverse-transcription controls. Data was collected on a 7300 Real-Time PCR System (Applied Biosystems). Oligonucleotides are provided in the Key Resource.

RNA expression profiling

RNA was purified from sorted mesenchymal cells using the NucleoSpin® RNA XS kit per manufacturer's recommendations (MACHEREY-NAGEL, Düren, Germany). Libraries were prepared using the Affymetrix WT Pico kit per manufacturer's recommendations (Affymetrix, Waltham, MA, USA) and then hybridized onto Affymetrix Clariom S arrays. The arrays were placed in a GeneChip Hybridization Oven 640 for 18 hours at 45°C, and then they were washed and stained in an Affymetrix Fluidics Station 450. The arrays were scanned using the Affymetrix GeneChip 7G 3000 Scanner. Data were analyzed on an Affymetrix GeneChip Command Console to generate CEL files. Affymetrix Transcriptome Analysis Console (TAC) software was used to treat the CEL files through Robust Multichip Analysis (RMA) algorithm, including probe-set signal integration, background correction and quantile normalization. TAC software also was used to identify differentially expressed genes. In this study, differentially expressed genes were defined by the following criteria: 1) false discovery rate (FDR) of ≤ 7.5%; 2) fold-change of ≥ 2; and 3) a minimum probe signal of 50 in the upregulated group. To identify the significantly enrichment of gene ontology (GO) terms and functional pathways, DAVID (http://david.abcc.ncifcrf.gov/tools.jsp), Kyoto Encyclopedia of Genes and Genomes (KEGG, http://www.genome.jp/kegg/), and pre-ranked Gene Set Enrichment Analysis (GSEA, http://software.broadinstitute.org/gsea/index.jsp) were used.

Mesenchymal stromal cell culture

Mice were sacrificed on embryonic day 16.5 (E16.5) or 1-4 days after birth, and hindlimb bones were harvested. Bones were mechanically disrupted in complete Dulbecco's modified eagle medium (DMEM) plus 20% fetal bovine serum (FBS) and penicillin-streptomycin using a mortar and pestle. The resulting cell suspension was cultured overnight at 37°C with 5% $CO_2$, and the following day non-adherent cells removed by gentle aspiration. Cells were cultured until reaching approximately 40% confluence (generally, 5-6 days). To induce adipogenesis, dexamethasone (100nM), insulin (5 µg/ml), and indomethacin (50 mM) were added to the culture media for 5 days. Where indicated, recombinant murine TGF-β (20ng/ml) and/or a combination of two MAPK inhibitors, U0126 (20 µM) and PD98059 (20 µM), were included in the cultures 24 hours prior to inducing adipogenesis.

Western Blot

Total cellular protein was isolated from culture mesenchymal cells using RIPA lysis buffer (#20-188, MilliporeSigma) inhibiting for protease and phosphatase (#78440, ThermoFisher). Twenty µg of protein was separated using pre-cast 4-15% gradient SDS gels (#456-1084, Bio-Rad) and transferred to nitrocellulose membranes (#IPVH00010.

Millipore). Membranes were incubated with a 1:1000 dilution of rabbit anti-PPARγ (PA3-821A, MilliporeSigma) or a 1:1000 dilution of rabbit anti-phospo (Ser82)-PPARγ) (#04-816, MilliporeSigma) in Tris buffered saline 100mM Tris HCl, 1.5M NaCl, pH 7.5 (TBS) (#P029, G-biosciences, Saint Louis, MO, USA) and 5% BSA (TBS) overnight at 4°C. Following multiple TBS washes, the membranes were incubated with a 1:5000 dilution of horseradish-peroxidase-conjugated anti-rabbit antibody (#A0545, MilliporeSigma) for 1 hour at room temperature. Following incubation with a chemiluminescence horse radish peroxidase substrate (Millipore, #WBKLS0100), the membranes were imaged using the Bio-Rad ChemiDoc XRS+ (BIO-RAD, Hercules, CA, USA.).

Quantification and Statistical Analysis

Significance was determined using PRISM software (GraphPad), except in the case of the RNA expression profiling data, which was analyzed using the Affymetrix Transcriptome Analysis Console. For single parameter analysis, unpaired t-test were used to assess statistical significance. For multiple parameter data, statistical significance was calculated using one-way or two-way analysis of variance (ANOVA). P values less than 0.05 were considered significant.

Supplemental References

Rodda, S. J. and A. P. McMahon (2006). "Distinct roles for Hedgehog and canonical Wnt signaling in specification, differentiation and maintenance of osteoblast progenitors." Development **133**(16): 3231-3244.

Madisen, L., T. A. Zwingman, S. M. Sunkin, S. W. Oh, H. A. Zariwala, H. Gu, L. L. Ng, R. D. Palmiter, M. J. Hawrylycz, A. R. Jones, E. S. Lein and H. Zeng (2010). "A robust and high-throughput Cre reporting and characterization system for the whole mouse brain." Nat Neurosci **13**(1): 133-140.

Leveen, P. (2002). "Induced disruption of the transforming growth factor beta type II receptor gene in mice causes a lethal inflammatory disorder that is transplantable." Blood **100**(2): 560-568.

Lu, Y., Y. Xie, S. Zhang, V. Dusevich, L. F. Bonewald and J. Q. Feng (2007). "DMP1-targeted Cre expression in odontoblasts and osteocytes." J Dent Res **86**(4): 320-325.

Nagasawa, T., S. Hirota, K. Tachibana, N. Takakura, S. Nishikawa, Y. Kitamura, N. Yoshida, H. Kikutani and T. Kishimoto (1996). "Defects of B-cell lymphopoiesis and bone-marrow myelopoiesis in mice lacking the CXC chemokine PBSF/SDF-1." Nature **382**(6592): 635-638.
