# Supplementary material for: TGF-β Signaling Plays an Essential Role in the Lineage Specification of Mesenchymal Stem/Progenitor Cells in Fetal Bone Marrow
Source: Stem Cell Reports. 2019 Jun 13;13(1):48–60. doi: 10.1016/j.stemcr.2019.05.017 (PMC6626889; doi:10.1016/j.stemcr.2019.05.017)
Supplement: Document S2. Article plus Supplemental Information [file mmc3.pdf]

Stem Cell Reports
Article

OPEN ACCESS

TGF-β Signaling Plays an Essential Role in the Lineage Specification of Mesenchymal Stem/Progenitor Cells in Fetal Bone Marrow

Grazia Abou-Ezzi,[1] Teerawit Supakorndej,[1] Jingzhu Zhang,[1] Bryan Anthony,[1] Joseph Krambs,[1] Hamza Celik,[1] Darja Karpova,[1] Clarissa S. Craft,[2] and Daniel C. Link[1,*]
[1]Division of Oncology, Washington University School of Medicine, 660 S. Euclid Avenue, Campus Box 8007, St. Louis, MO 63110, USA
[2]Division of Bone and Mineral Diseases, Washington University, St. Louis, MO, USA
*Correspondence: danielclink@wustl.edu
https://doi.org/10.1016/j.stemcr.2019.05.017

SUMMARY

Mesenchymal stromal cells are key components of hematopoietic niches in the bone marrow. Here we abrogated transforming growth factor β (TGF-β) signaling in mesenchymal stem/progenitor cells (MSPCs) by deleting *Tgfbr2* in mesenchymal cells using a doxycycline-repressible *Sp7* (osterix)-Cre transgene. We show that loss of TGF-β signaling during fetal development results in a marked expansion of CXCL12-abundant reticular (CAR) cells and adipocytes in the bone marrow, while osteoblasts are significantly reduced. These stromal alterations are associated with significant defects in hematopoiesis, including a shift from lymphopoiesis to myelopoiesis. However, hematopoietic stem cell function is preserved. Interestingly, TGF-β signaling is dispensable for the maintenance of mesenchymal cells in the bone marrow after birth under steady-state conditions. Collectively, these data show that TGF-β plays an essential role in the lineage specification of fetal but not definitive MSPCs and is required for the establishment of normal hematopoietic niches in fetal and perinatal bone marrow.

INTRODUCTION

The bone marrow microenvironment is uniquely adapted to support hematopoiesis. A complex network of stromal cells in the bone marrow provides key signals that support the proliferation and survival of hematopoietic stem/progenitor cells (Calvi and Link, 2015). CXCL12-abundant reticular (CAR) cells are perivascular mesenchymal stromal cells that express high levels of CXCL12 and stem cell factor (Sugiyama et al., 2006); they overlap considerably with Leptin-receptor$^+$ stromal cells (Zhou et al., 2014) and Nestin-GFP$^+$ stromal cells in the bone marrow (Mendez-Ferrer et al., 2010). CAR cells and NG2$^+$ arteriolar pericytes produce cytokines and chemokines that play crucial roles in regulating hematopoietic stem cells (HSCs), including CXCL12 and stem cell factor (Ding and Morrison, 2013; Ding et al., 2012; Greenbaum et al., 2013; Kunisaki et al., 2013). Adipocytes are rare in the bone marrow at birth but increase with aging and after myeloablation (Justesen et al., 2001; Zhou et al., 2017). The presence of adipocytes in the bone marrow negatively correlates with hematopoietic activity (Naveiras et al., 2009). However, a recent study showed that adipocytes promote hematopoietic recovery following myeloablation through production of stem cell factor (Zhou et al., 2017), suggesting a context-specific role for adipocytes in regulating hematopoiesis.

The development and maintenance of mesenchymal stromal cells in the bone marrow is not well characterized. Lineage tracing studies show that *Sp7* (*osterix*)-*cre*-targeted mesenchymal stem/progenitor cells (MSPCs) are present in the perichondrium of the future hindlimb by embryonic day 12.5 (E12.5) (Logan et al., 2002; Maes et al., 2010). These fetal MSPCs transiently give rise to all mesenchymal stromal cells in the bone marrow, including osteoblasts, CAR cells, arteriolar pericytes, and adipocytes. However, these stromal cells are gradually replaced during adulthood. Indeed, a distinct *osterix-Cre*-targeted MSPC population is present in neonatal bone marrow and gives rise to long-lived mesenchymal stromal cells (Mizoguchi et al., 2014). The signals regulating lineage specification of MSPCs also are not well characterized. Omatsu and colleagues, in two separate studies, showed that *Foxc1* and *Ebf1/Ebf3* contribute to the lineage specification of postnatal MSPCs. Specifically, the Foxc1 transcription factor negative regulates adipocyte differentiation of postnatal MSPCs, while the Ebf1/Ebf3 transcription factors inhibit osteoblast differentiation (Omatsu et al., 2014; Seike et al., 2018).

Transforming growth factor β (TGF-β) is an inflammatory cytokine that also may contribute to MSPC differentiation. Cell culture studies show that TGF-β negatively regulates adipocyte and terminal osteoblast differentiation, while stimulating osteoblast progenitor proliferation (Alliston et al., 2001; Ignotz and Massague, 1985; Sparks et al., 1992). Studies examining the role of TGF-β signaling in MSPC differentiation *in vivo* are limited. Loss of *Tgfb1* is associated with bone loss and a deficiency of osteoblasts (Tang et al., 2009). *Tgfbr2*, encoding TGF-β receptor 2, is required for TGF-β signaling. Deletion of *Tgfbr2* using *Prx1-Cre*, which is active in early limb bud mesenchyme, results in severe skeletal defects and embryonic lethality (Seo and Serra, 2007). Wang et al. (2013) used an osterix-Cre

 © 2019 The Author(s).
This is an open access article under the CC BY license (http://creativecommons.org/licenses/by/4.0/).

(*Osx-Cre*) transgene to delete *Tgfbr2* in mesenchymal progenitors. They showed that Osx-Cre, Tgfbr2$^{fl/fl}$ mice have impaired tooth development and reduced mineralization of the mandible due to reduced osteoblast differentiation. In humans, genetic alterations leading to enhanced TGF-β signaling are associated with bone dysplasia in Camurati-Engelmann disease (Wallace and Wilcox, 1993). Of note, TGF-β regulates HSC quiescence and hematopoietic recovery following myeloablation (Brenet et al., 2013; Yamazaki et al., 2011; Zhao et al., 2014). Whether TGF-β signaling in mesenchymal stromal cells contributes to these hematopoietic responses is an open question.

In this study, we characterize the contribution of TGF-β signaling in MSPCs on the development of mesenchymal stromal cells that comprise the bone marrow hematopoietic niche. We show that loss of TGF-β signaling in *Osx-Cre*-targeted fetal MSPCs results in alterations in mesenchymal stromal cells, including marked expansions of CAR cells and adipocytes. Both canonical and noncanonical TGF-β signaling in fetal MSPCs contribute to this phenotype. The resulting alterations in mesenchymal stromal cells are associated with a reduced capacity to support HSCs and a shift in hematopoiesis from lymphopoiesis to myelopoiesis. Together, these data suggest that TGF-β plays a key role in the lineage specification of MSPCs and is required for the emergence of a normal hematopoietic niche during fetal bone marrow development.

RESULTS

Deletion of *Tgfbr2* in *Osx-Cre*-Targeted Mesenchymal Cells Results in a Loss of Mature Osteoblasts

To investigate the role of TGF-β in the development and maintenance of bone marrow mesenchymal stromal cells, we deleted *Tgfbr2* in mesenchymal cells using a doxycycline-repressible *Sp7* (osterix)-Cre transgene (*Osx-Cre*) (Maes et al., 2010). Previous studies have shown that *Osx-Cre* targets most mesenchymal stromal cells in the bone marrow, including osteoblasts, adipocytes, pericytes, and CAR cells, but not endothelial cells or hematopoietic cells. *Osx-Cre, Tgfbr2$^{fl/fl}$* male and female mice are severely runted with a body weight less than 30% that of littermate controls (Figures 1A and 1B). Since most *Osx-Cre, Tgfbr2$^{fl/fl}$* died by 4 weeks of age, we focused our initial analysis of mice at 3 weeks of age, when they appeared healthy.

The severe runting in *Osx-Cre, Tgfbr2$^{fl/fl}$* mice suggested impaired bone development. Indeed, micro-computerized tomography (micro-CT) (Bouxsein et al., 2010) analysis of male mice at 3 weeks of age showed significant reductions in the bone volume and bone mineral density in trabecular bone and a decrease in bone thickness and bone area in cortical bone (Figure 1C). An increase in bone marrow trabecularization also was observed by micro-CT (Figure 1D) and in histological sections (Figure S1A). Increased trabecularization of the bone marrow can be seen with impaired osteoclast activity. However, the serum level of C-terminal telopeptide of type I collagen, a measure of bone resorption (Bonde et al., 1995), was similar in *Osx-Cre, Tgfbr2$^{fl/fl}$* mice and control mice (Figure S1D). Moreover, the number of TRAP^{+} osteoclasts and mRNA expression in the bone marrow of the osteoclast-specific genes, *Acp5* (TRAP) and *Ctsk* (cathepsin K), was comparable with control mice (Figures S1B and S1C). Thus, altered osteoclast function is not responsible for the bony defects in *Osx-Cre, Tgfbr2$^{fl/fl}$* mice.

We next examined osteoblasts using histomorphometry of bone sections from *Osx-Cre, Tgfbr2$^{fl/fl}$ Ai9* mice; these mice express tdTomato in all mesenchymal bone marrow stromal cells, including osteoblasts. The number of tdTomato^{+} endosteal cells in the bone marrow of *Osx-Cre, Tgfbr2$^{fl/fl}$ Ai9* mice was reduced approximately 4-fold compared with control mice (Figures 1E and 1F). Consistent with this finding, we also observed a consistent loss of osteocalcin^{+} cells along the endosteum (Figure 1G).

There is in evidence from cell culture systems that TGF-β signaling in osteoblast progenitors negatively regulates terminal osteoblast differentiation *in vitro*. To examine this *in vivo*, we generated DMP1-Cre, Tgfbr2$^{fl/fl}$ mice. We previously showed that the *DMP1-Cre* transgene targets mature osteoblasts and a subset of CAR cells that likely includes osteoblast progenitors (Zhang and Link, 2016). *DMP1-Cre, Tgfbr2$^{fl/fl}$* mice are not runted. Moreover, the number of osteocalcin^{+} endosteal osteoblasts is normal (Figure 1H), suggesting that TGF-β signaling is not required for terminal osteoblast differentiation. Together, these data suggest that TGF-β signaling in a mesenchymal progenitor is required for the efficient development of mature osteoblasts.

Deletion of *Tgfbr2* in *Osx-Cre*-Targeted Mesenchymal Cells Results in a Marked Increase in Bone Marrow Adipocytes

Previous studies showed that TGF-β negatively regulates the adipogenic differentiation of mesenchymal cell lines *in vitro* (Choy and Derynck, 2003). We used osmium tetroxide staining with micro-CT to visualize and quantify bone marrow adiposity in *Osx-Cre, Tgfbr2$^{fl/fl}$* mice at 3 weeks of age, when bone marrow adiposity should be minimal (Scheller et al., 2014). As expected in control mice, little osmium staining was observed, mainly in the diaphyseal region (Figure 2A). In contrast, osmium staining was seen throughout the femur, and was increased nearly 80-fold in *Osx-Cre, Tgfbr2$^{fl/fl}$* mice (Figures 2A and 2B). Consistent with this finding, a marked increase in oil red^{+} cells in the bone marrow of *Osx-Cre, Tgfbr2$^{fl/fl}$* mice also was observed

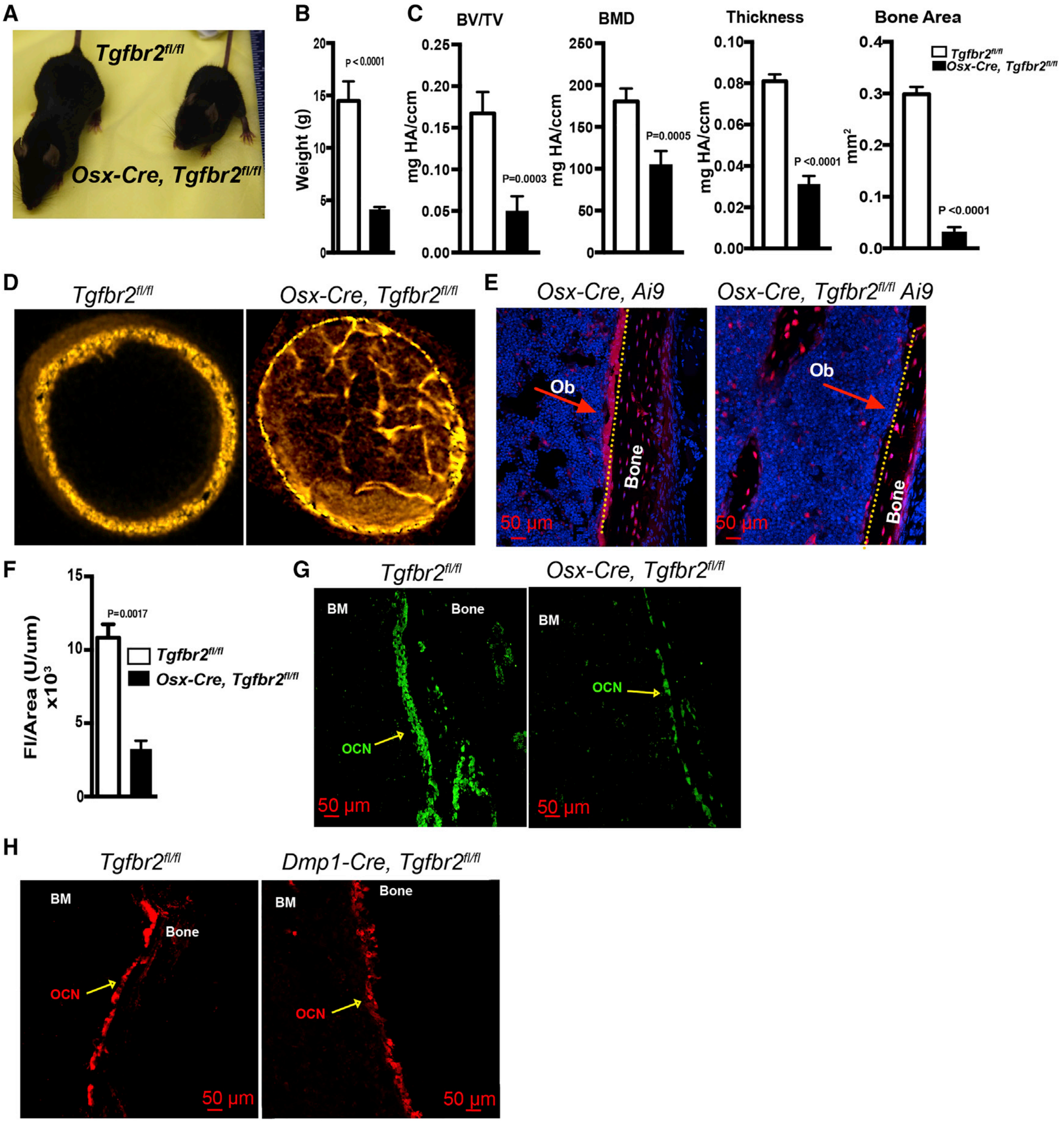

Figure 1. Loss of TGF-β Signaling in Mesenchymal Cells Inhibits Osteoblast Maturation

(A) *Osx-Cre* Tgfbr2$^{fl/fl}$ mouse and a littermate control mouse.

(B) Body weight at 3 weeks of age (n = 5).

(C) Micro-computed tomography (micro-CT) data showing bone volume density (BV/TV), bone mineral density (BMD), thickness, and bone area (n = 4).

(D) Three-dimensional reconstruction of the micro-CT data in the diaphyseal region of a femur.

(E) Representative photomicrographs of the diaphyseal region of *Osx-Cre, Ai9*, and *Osx-Cre Tgfbr2$^{fl/fl}$ Ai9* mice showing TdTomato^{+} osteoblasts (OB) lining the bone surface. Counterstaining with DAPI (blue) highlights nuclei.

(F) Quantification of endosteal TdTomato^{+} osteoblasts shown as fluorescence intensity per unit of bone surface area (n = 3).

(G) Representative photomicrographs showing osteocalcin expression (green).

(H) Representative photomicrographs showing osteocalcin expression (red) in femurs from *Tgfbr2$^{fl/fl}$* and *Dmp-1-Cre, Tgfbr2$^{fl/fl}$* mice. Original magnification 20× for all images. Data represent the means ± SEM.

(Figure 2C). Finally, expression of several genes associated with adipocyte differentiation was significantly increased in the bone marrow of *Osx-Cre, Tgfbr2$^{fl/fl}$* mice, including peroxisome proliferator-activated receptor gamma (*Pparg*) and fatty acid binding protein 4 (*Fabp4*) (Figure 2D). Collectively, these data show that loss of TGF-β signaling in

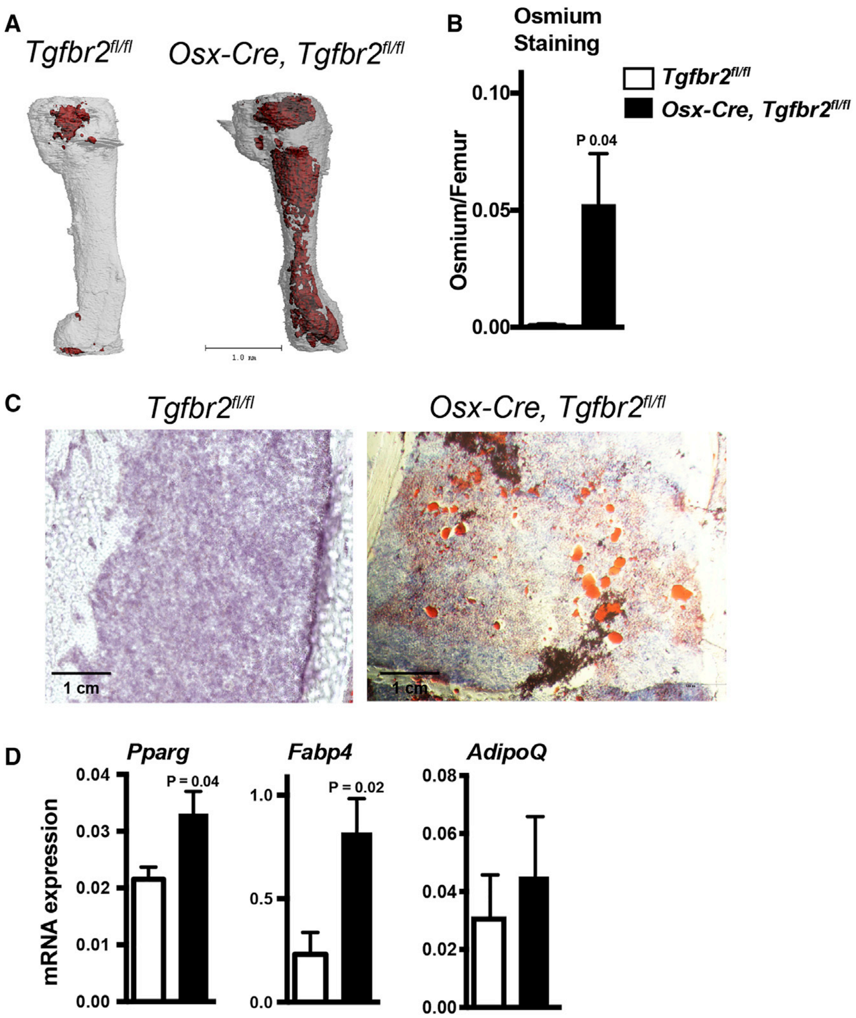

Figure 2. Loss of TGF-β Signaling in Mesenchymal Cells Is Associated with Increased Bone Marrow Adiposity

(A) Representative micro-CT images showing osmium tetraoxide staining of femurs.

(B) Quantification of the osmium tetraoxide signal (n = 5).

(C) Representative images of femur sections stained with oil red (red). Original magnification, 10×.

(D) RNA expression of the indicated gene relative to β-actin mRNA is shown (n = 5–7). Data represent the means ± SEM.

mesenchymal cells results in a massive increase in bone marrow adiposity.

Deletion of *Tgfbr2* in *Osx-Cre*-Targeted Mesenchymal Cells Results in an Expansion of CAR Cells

The signals that regulate the development and maintenance of CAR cells are largely unknown. To assess the impact of TGF-β signaling on CAR cell development and/or maintenance, we generated *Osx-Cre, Tgfbr2$^{fl/fl}$ Cxcl12^{gfp}* mice. As expected, in control mice, CXCL12-GFP-bright perivascular CAR cells were seen throughout the bone marrow (Figure 3B). In *Osx-Cre, Tgfbr2$^{fl/fl}$ Cxcl12^{gfp}* mice the number of CAR cells was increased. Indeed, by histomorphometry, CAR cell number was increased nearly 7-fold compared with control mice, and GFP expression per CAR cell was increased (Figures 3B and 3C). Of note, the increase in CAR cells was not due to aberrant CXCL12-GFP expression in adipocytes, since perilipin^{+} adipocytes did not overlap with CXCL12-GFP bright (CAR) cells (Figure S2). Despite the increase in CAR cell number, total bone marrow CXCL12 mRNA expression was significantly decreased, suggesting that CXCL12 mRNA expression per CAR cell is reduced (Figure 3A). Indeed, despite the increase in CXCL12-GFP expression (Figure 3B), a trend to decreased CXCL12 mRNA expression (but not other niche factors) was observed in CAR cells sorted from *Osx-Cre, Tgfbr2$^{fl/fl}$ Cxcl12^{gfp}* mice, suggesting that the mechanisms regulating CXCL12-GFP expression are distinct from those regulating endogenous *Cxcl12* expression (Figure 3D). These data show that loss of TGF-β signaling in mesenchymal cells results in an expansion of CAR cells that have modestly reduced CXCL12 expression.

Deletion of *Tgfbr2* in *Osx-Cre*-Targeted Mesenchymal Cells Is Associated with Altered Hematopoiesis

We next examined the effect of the altered bone marrow microenvironment in *Osx-Cre, Tgfbr2$^{fl/fl}$* mice on hematopoiesis. Compared with controls, *Osx-Cre, Tgfbr2$^{fl/fl}$* mice displayed pancytopenia, with significant decreases in the level of circulating neutrophils, B cells, and T cells

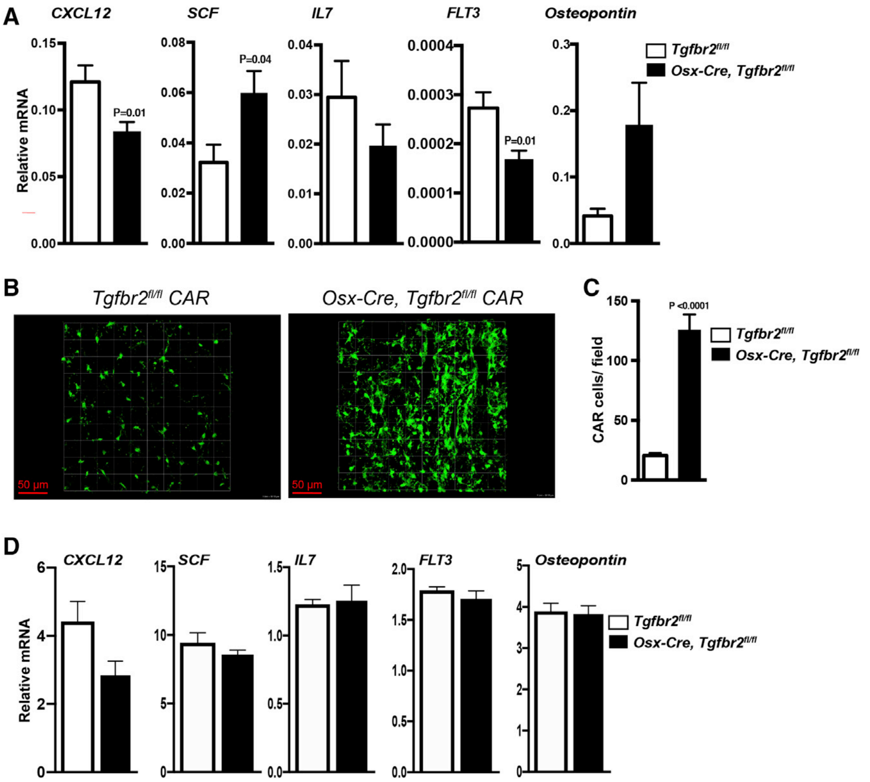

Figure 3. Loss of TGF-β Signaling in Mesenchymal Cells Is Associated with an Expansion of CAR Cells

(A) Total bone marrow RNA expression of the indicated gene relative to β-actin mRNA is shown (n = 5).

(B) Representative images of femur sections showing GFP bright (green) CAR cells. Original magnification 20×.

(C) Quantification of CAR cells (n = 4).

(D) CXCL12-GFP-bright lineage$^-$ cells were sorted from the bone marrow of *Osx-Cre Tgfbr2*$^{fl/fl}$ *Cxcl12*^{gfp} or *Tgfbr2*$^{fl/fl}$ *Cxcl12*^{gfp} mice. RNA expression of the indicated gene relative to β-actin mRNA is shown (n = 4–6). Data represent the means ± SEM.

(Figures 4A and 4B). Furthermore, bone marrow and spleen cellularity were reduced, even after normalizing to body weight (Figures 4C–4F). In the bone marrow, a shift from lymphopoiesis to myelopoiesis was observed, characterized by a marked decrease in B cells and a modest increase, on a percentage basis, in myeloid cells and granulocyte/macrophage progenitors, although the absolute number of each hematopoietic cell population was reduced (Figures 4G, 4H, and S3A–S3C). On a percentage basis, phenotypic HSCs (Kit$^+$Sca1$^+$lineage$^-$CD150$^+$CD48$^-$ cells) were reduced approximately 2-fold (Figures 4I and S3D). Due to bone marrow hypocellularity, this resulted in a marked (approximately 10-fold) decrease in the total number of HSCs per femur (Figure 4J). Even after adjusting for bone marrow volume (total volume − bone volume), the total number of phenotypic HSCs was reduced 2.8-fold in *Osx-Cre, Tgfbr2*$^{fl/fl}$ mice (Figure 4K). Of note, the number of Kit$^+$Sca1$^+$lineage$^-$ cells in the spleen of *Osx-Cre, Tgfbr2*$^{fl/fl}$ mice was markedly reduced, arguing against a migration of hematopoietic progenitors from the bone marrow to spleen (Figures S3E–S3H).

To assess HSC function, competitive repopulation assays were performed using unpurified bone marrow cells. Despite the decrease in phenotypic HSCs, multilineage long-term repopulating activity of *Osx-Cre, Tgfbr2*$^{fl/fl}$ bone marrow was comparable with control mice (Figure 4L). Moreover, similar donor engraftment was observed following secondary transplantation, suggesting that HSC self-renewal is normal (Figure 4M). Collectively, these data show that TGF-β signaling in mesenchymal progenitors is required for the development of a normal hematopoietic niche in the bone marrow. Specifically, the altered bone marrow microenvironment in *Osx-Cre, Tgfbr2*$^{fl/fl}$ mice is associated with a reduced capacity to support hematopoiesis (in particular, B lymphopoiesis), but with relatively preserved ability to support HSCs under basal conditions.

Postnatal Deletion of *Tgfbr2* in *Osx-Cre*-Targeted Mesenchymal Cells Does Not Result in Impaired Osteoblast or Adipocyte Development

A recent study suggested that mesenchymal stromal cells in the bone marrow are derived from two distinct types of MSPCs (Mizoguchi et al., 2014). Primitive or fetal MSPCs, defined as *Osx-Cre*-targeted cells present in fetal bone on E12.5, are responsible for osteoblasts and CAR cells through approximately 3 weeks after birth. Definitive or postnatal MSPCs, defined as *Osx-Cre*-targeted cells present at birth, are responsible for the generation of osteoblasts, CAR, and adipocytes cells in adult mice. To investigate the contribution of TGF-β signaling in the lineage specification of definitive MSPCs, we characterized bone marrow stromal cells in which *Tgfbr2* was deleted postnatally by removing doxycycline at birth. Consistent with a previous

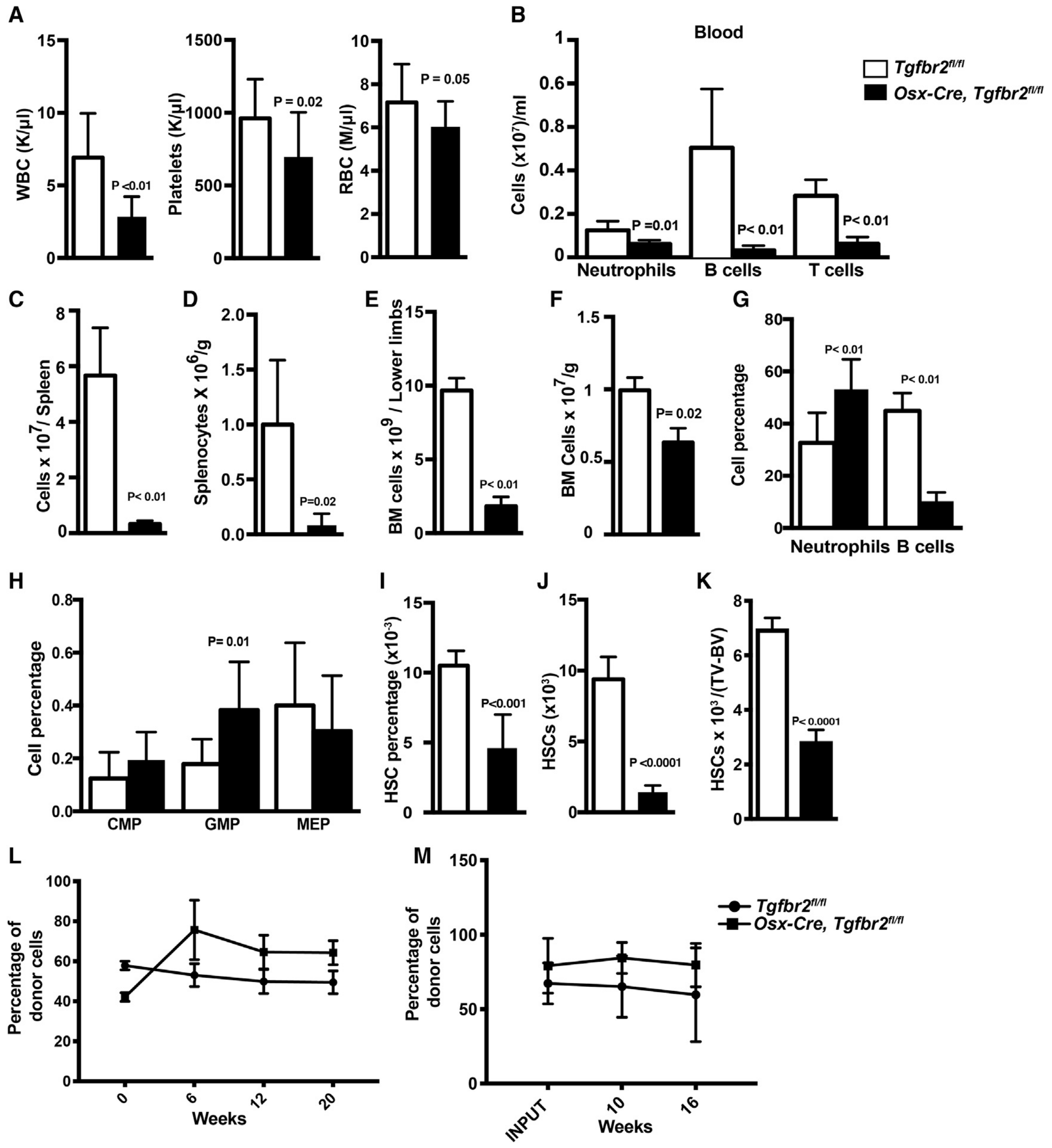

Figure 4. Loss of TGF-β Signaling in Mesenchymal Cells Results in Perturbed Hematopoiesis

(A) Peripheral blood counts. WBC, white blood cells; RBC, red blood cells (n = 12).

(B) Number of neutrophils, B cells, and T cells in the blood is shown (n = 4).

(C–F) Spleen and bone marrow (BM) cellularity (per pelvis and combined lower limbs) (n = 4) (C and E) and after adjusting for body weight (n = 4) (D and F).

(G–I) Percentage of the indicated cell type in the bone marrow is shown (n = 5): neutrophils and B cells (G), CMP (common myeloid progenitor), GMP (granulocytes-macrophages progenitors), MEP (myeloid-erythrocytes progenitors) (H), and HSCs (c-kit$^+$, lineage$^-$, Sca1$^+$, CD150$^+$, CD48$^-$ cells) (I).

(J and K) Shown is the absolute number of HSCs per pelvis and combined lower limbs (J) and HSC number after correcting for bone marrow volume (K), which was estimated using micro-CT data as total volume (TV) of femur minus bone volume (BV) (n = 4).

(L) Competitive repopulation assays were performed with a 1:1 ratio of donor and wild-type competitor bone marrow. Shown is the percentage of donor (Ly5.2) cells in peripheral blood (n = 15, from three independent experiments).

(M) Secondary transplantation was performed after 24 weeks. Shown is the percentage of donor (Ly5.2) cells in the secondary recipients (n = 5). Data represent the means ± SEM.

study, lineage tracing using *Osx-Cre Tgfbr$^{fl/fl}$ Ai9* mice suggests that induction of *Osx-Cre* expression on postnatal day 0 (P0) results in efficient targeting of osteoblasts and CAR cells (Figure 5A) (Mizoguchi et al., 2014). *Osx-Cre, Tgfbr2$^{fl/fl}$* mice with postnatal *Tgfbr2* deletion were of normal size. Bone marrow cellularity was comparable (Figure S4D) with control mice, and no increase in bone marrow trabeculae was observed (Figure 5B). Osteoblast number, as measured by osteocalcin staining, was comparable with control mice (Figure 5C). Moreover, no increase in perilipin$^+$ cells was observed (Figure 5D). Consistent with the lack of stromal changes, postnatal deletion of *Tgfbr2* had no effect on basal hematopoiesis (Figures S4A–S4G). Collectively, these data suggest that TGF-β signaling in definitive MSPCs is dispensable for lineage specification in the early postnatal period.

TGF-β Signaling in Embryonic *Osx-Cre*-Targeted Mesenchymal Progenitors Contributes to Osteoblast Lineage Specification

The lack of major perturbations in mesenchymal stromal cells in the bone marrow of postnatally deleted *Osx-Cre, Tgfbr2$^{fl/fl}$* mice suggested that TGF-β signaling in fetal MSPCs may contribute to lineage specification. To explore this possibility, we examined endochondral bone development in the hind limbs of *Osx-Cre, Tgfbr2$^{fl/fl}$ Ai9* mice maintained off doxycycline, which results in constitutive *Osx-Cre* expression. We first examined mice at E14.5, at a time just before the development of primary ossification centers. As reported previously, at this time point, the majority of *Osx-Cre*-targeted (tdTomato$^+$) cells localize to the perichondrium that surrounds hypertrophic chondrocytes at the site of future long bones (Maes et al., 2010) (Figures 6A and 6B). Maes et al. showed that these osterix-expressing cells invade the cartilage and give rise to trabecular osteoblasts and other bone marrow stromal cells. The number and perichondrial localization of *Osx-Cre*-targeted cells in *Osx-Cre, Tgfbr2$^{fl/fl}$ Ai9* mice was similar to control mice (Figure 6B). Strikingly, even at this early stage of bone development, a marked increase in perilipin$^+$ cells was observed in the bone collar of the developing hindlimb at E14.5 of *Osx-Cre, Tgfbr2$^{fl/fl}$* mice (Figure 6C). The increase in perilipin$^+$ cells in *Osx-Cre, Tgfbr2$^{fl/fl}$* mice on E16.5 was even more striking, with most Osx-Cre-targeted cells also staining for perilipin (Figures 6D and S5D). However, *Osx-Cre, Tgfbr2$^{fl/fl}$* chondrocyte number and distribution were similar to littermate controls (Figures S5A and S5B).

To further assess the impact of TGF-β signaling on fetal MSPC lineage specification, we sorted *Osx-Cre*-targeted (TdTomato$^+$) cells from the hind limbs of E16.5 *Osx-Cre, Tgfbr2$^{fl/fl}$ Ai9* or *Osx-Cre Ai9* mice and performed RNA expression profiling. Gene set enrichment analysis yielded multiple hits for increased adipogenesis (Figure 6E). Indeed, expression of key regulators or markers of adipogenesis, including *Pparγ*, *Plin2* (perilipin), *Cd36*, *Fabp4*, and *Adipoq* (adiponectin) was increased in *Osx-Cre, Tgfbr2$^{fl/fl}$ Ai9* cells (Figure 6F). Interestingly, no alteration in expression of osteoblast lineage genes was observed (Figure S5C). Together, these data suggest that TGF-β signaling plays a key role in lineage specification of fetal MSCPs, suppressing adipogenesis while supporting osteoblast development.

Noncanonical and Canonical TGF-β Negatively Regulates Adipogenesis

Canonical TGF-β signaling depends on SMAD4 (Massagué, 2012). To investigate whether MSPC lineage specification by TGF-β depends on SMAD4, we generated *Osx-Cre, Smad4$^{fl/fl}$* mice. Of note, canonical signaling by TGF-β and other TGF family members, such as bone morphogen proteins and activins, are disrupted in these mice (Miyazawa et al., 2002). *Osx-Cre, Smad4$^{fl/fl}$* mice are runted to a similar degree as *Osx-Cre, Tgfbr2$^{fl/fl}$* mice. Also similar to *Osx-Cre, Tgfbr2$^{fl/fl}$* mice, *Osx-Cre Smad4$^{fl/fl}$* mice displayed increased trabecularization of their bone marrow cavity and a loss of mature osteoblasts (Figures 7A and 7B). However, the magnitude of the increase in bone marrow adiposity was reduced in *Osx-Cre Smad4$^{fl/fl}$* mice compared with *Osx-Cre, Tgfbr2$^{fl/fl}$* mice as assessed by perilipin and oil red staining (Figures 7C, 7D, and S6).

These data suggested that noncanonical signaling contributes to the suppressive effect of TGF-β on adipogenesis. To test this hypothesis, we generated cultures of mesenchymal stromal cells from wild-type neonatal bone marrow (Figure 7E). Adipogenesis was induced by the inclusion of dexamethasone, insulin, and indomethacin in the culture media (CFU-A). As expected, in wild-type cultures, the addition of TGF-β potently suppressed adipocyte formation, as measured by oil red staining (Figure 7F). Of note, similar results were observed with mesenchymal stromal cells derived from E16.5 hindlimbs (Figure S7). Noncanonical TGF-β signaling includes activation of mitogen-activated protein kinase (MAPK) (Zhang, 2009). To assess the role of MAPK activation on the suppression of adipogenesis by TGF-β, we pharmacologically inhibited MAPK activation in wild-type MSPC cultures. Inhibition of MAPK alone did not suppress adipocyte formation. However, it completely blocked the suppressive effect of TGF-β on adipogenesis (Figure 7F). Previous studies showed that phosphorylation of serine 82 of PPARγ by MAPK decreases its transcriptional activity (Camp and Tafuri, 1997). Since PPARγ is a master regulator of adipogenesis, we assessed the ability of TGF-β to induce PPARγ phosphorylation. Indeed, the addition of TGF-β to the MSPC cultures resulted in reproducible PPARγ phosphorylation (Figures 7H and 7E). Collectively, these data suggest that TGF-β suppresses

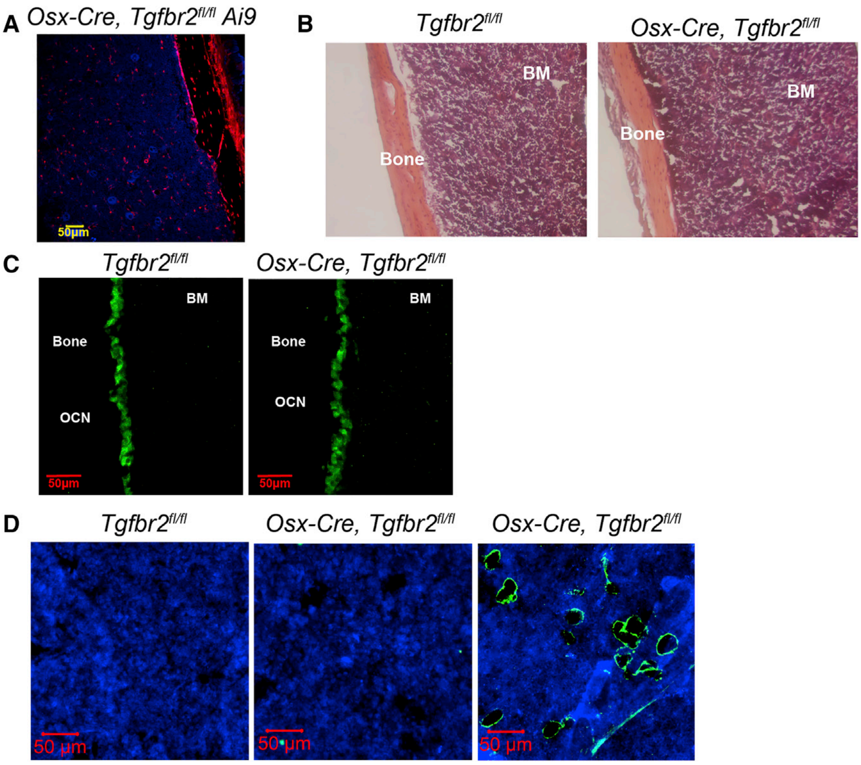

Figure 5. TGF-β Signaling in Mesenchymal Cells Postnatally Is Not Required for Osteoblast and Adipocyte Development

Doxycycline was removed at birth (postnatal day) from Osx-Cre *Tgfbr2*$^{\Delta/\Delta}$ or control mice to activate the *Osx-Cre* transgene.

(A) Representative image of *Osx-Cre Tgfbr2*$^{fl/fl}$, *Ai9* femur sections showing TdTomato (red) and DAPI (blue staining).

(B) Representative images of H&E-stained femur sections.

(C) Representative images of osteocalcin (green)-stained femur sections.

(D) Representative images of femur sections stained for perilipin (green) and DAPI (blue). The far right panel shows a femur section from an *Osx-Cre Tgfbr2*$^{fl/fl}$ mouse maintained off doxycycline during development, resulting in constitutive *Osx-Cre* expression. Original magnification 20×.

adipocyte specification of MSPCs, in part, in an MAPK-dependent fashion through phosphorylation of PPARγ.

DISCUSSION

In this study, we show that TGF-β signaling plays a key role in the lineage specification of MSPCs during fetal bone development. Specifically, loss of TGF-β signaling in *Osx-Cre*-targeted MSPCs at sites of developing bones results in a marked expansion of adipocytes and CAR cells, while mature osteoblasts are reduced. In contrast, deletion of *Tgfbr2* in *Osx-Cre*-targeted MSPCs at birth has no apparent effect on mesenchymal cell lineage commitment. These data suggest that TGF-β signaling in fetal, but not adult, MSPCs, plays a key role in lineage specification. Of note, the ability of TGF-β to suppress adipogenesis in cultures MPSCs from fetal or postnatal day 1–4 bone marrow was similar, suggesting that non-cell intrinsic mechanisms may be responsible for the differential reliance on TGF-β *in vivo*. Whether differences in the level of active TGF-β or the presence of other TGF-β family members in the local microenvironment account for this difference will require further study. Likewise, further study is needed to define the importance of TGF-β signaling on the response of bone marrow resident MSPCs to aging or stressors, such as bone fracture or myeloablative therapy.

Previous studies have yielded mixed results with respect to the role of TGF-β signaling in the regulation of osteoblasts (Alliston et al., 2001; Wu et al., 2016). Cell culture studies suggested that TGF-β negatively regulates terminal osteoblast differentiation (Alliston et al., 2001). Consistent with these data, abrogation of TGF-β signaling in osteoblasts and osteoblast precursors in *Ocn-Cre Tgfbr2*$^{fl/fl}$ mice is associated with an increased bone mass (Qiu et al., 2010). However, our data show that constitutive deletion of *Tgfbr2* in MSPCs is associated with reduced bone mass and a loss of osteoblasts. Moreover, we observed no change in osteoblast number in *DMP1-Cre Tgfbr2*$^{fl/fl}$ mice, in which TGF-β signaling is abrogated in mature osteoblasts and pre-osteoblasts (Zhang and Link, 2016). Although we did not directly measure bone metabolism, these data suggest that TGF-β signaling is dispensable for terminal osteoblast differentiation. Of note, our data are consistent with a previous report showing that global loss of *Tgfb1* is associated with bone loss (Tang et al., 2009). Reconciling these disparate results is uncertain, but may be related to stage-specific effects of TGF-β signaling on osteoblast development.

The mechanisms regulating MSPC differentiation into adipocytes are not well understood. Our data show that TGF-β is a potent negative signal regulating adipocyte specification in the developing bone marrow. Indeed, in the absence of TGF-β signaling in fetal MSPCs, there is a striking increase in bone marrow adiposity. This phenotype is

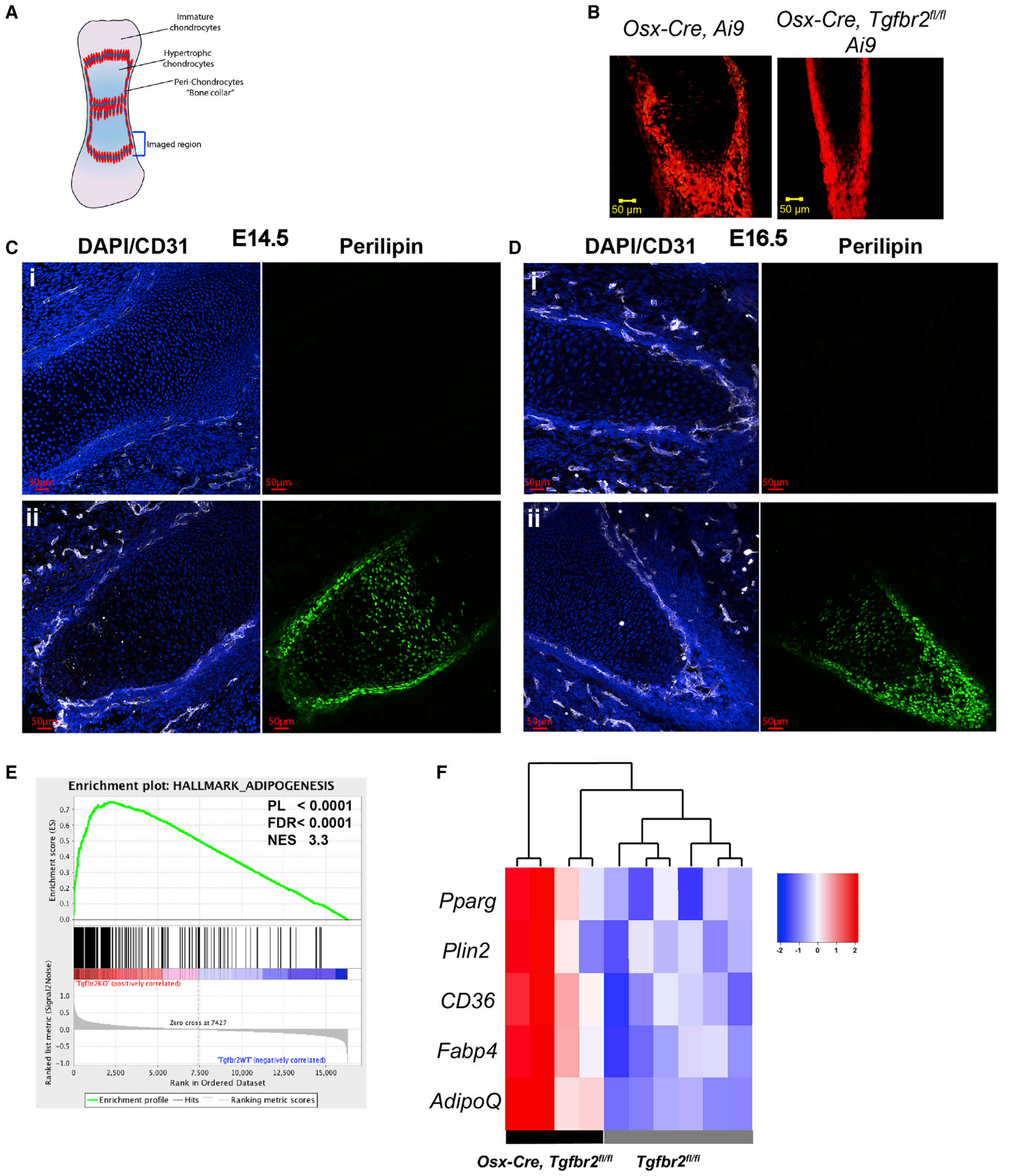

Figure 6. TGF-β Signaling Is Required for the Lineage Specification of Fetal Mesenchymal Stem/Progenitor Cells

(A) Schematic of bone development at E14.5 showing the perichondrial "bone collar" (red) surrounding hypertrophic chondrocytes (light blue). The region imaged in (B) is shown.

(B) Representative images of E14.5 hindlimb sections showing TdTomato^{+} cells comprising the perichondrial collar. Original magnification 20×.

(legend continued on next page)

similar to that reported for mice whereby the transcription factor *Foxc1* is deleted in mesenchymal progenitors (Omatsu et al., 2014). However, we observed no difference in *Foxc1* mRNA expression in sorted fetal MSPCs from *Osx-Cre, Tgfbr2*$^{fl/fl}$ versus control mice (gene expression normalized signal: 52.0 ± 6.0 versus 60.3 ± 7.3, respectively; $p = 0.41$). Previous studies have suggested that TGF-β inhibits adipogenesis in cell lines in an SMAD-dependent fashion through repression of CCAAAT/enhancer binding protein transcriptional activity, which ultimately results in reduced PPARγ mRNA expression (Alliston et al., 2001). Consistent with this observation, we observed increased PPARγ mRNA in sorted fetal MSPCs from Osx-Cre, Tgfbr2$^{fl/fl}$ mice. Furthermore, our data show that SMAD-independent signaling also contributes to the inhibition of adipogenesis by TGF-β. Consistent with previous studies, we show that activation of MAPK by TGF-β results in phosphorylation of serine-82 of PPARγ, which is known to inhibit its transcriptional activity (Adams et al., 1997; Camp and Tafuri, 1997; Han et al., 2000). Indeed, we show that inhibition of MAPK abrogates the ability of TGF-β to suppress adipocyte differentiation of culture primary MSPCs. Together, these data suggest that both canonical and noncanonical TGF-β signaling contribute to the lineage specification of MSPC in the bone marrow.

Our data suggest that TGF-β signaling plays an important role in establishing hematopoietic niches in the bone marrow. Significant alterations in three important stromal components of the niche are altered in *Osx-Cre, Tgfbr2*$^{fl/fl}$ mice. CAR cells, a key component of the stem cell niche, are increased approximately 5-fold. However, despite the increase in CAR cells, total bone marrow expression of CXCL12 was modestly decreased. This can be explained, in part, by reduced CXCL12 mRNA expression in CAR cells, although loss of CXCL12 expression from other stromal cell populations, such as osteoblasts, likely contributes to the overall decrease in bone marrow CXCL12. Although phenotypic HSCs are modestly reduced, functional HSCs, as measured by long-term repopulating assays, were normal after adjusting for bone marrow cellularity. Consistent with previous studies suggesting that osteoblasts are a key component of the lymphoid niche in the bone marrow (Ding and Morrison, 2013; Yu et al., 2016), a prominent shift from lymphopoiesis to myelopoiesis was observed in *Osx-Cre, Tgfbr2*$^{fl/fl}$ mice. Increased bone marrow adiposity is associated with reduced hematopoietic activity. Consistent with this observation, we observed reduced bone marrow cellularity in *Osx-Cre, Tgfbr2*$^{fl/fl}$ mice (even after adjusting for their reduced size). A recent study showed that adipocytes are induced following myeloablation and contribute to hematopoietic recovery (Zhou et al., 2017). TGF-β also is induced, raising the possibility that increased TGF-β signaling may suppress adipocyte expansion following myeloablation and limit hematopoietic recovery.

In summary, our data suggest that TGF-β plays a key role in the lineage specification of fetal MSPCs during development and is required for the proper development of fetal hematopoietic niches in the bone marrow. The contribution of TGF-β signaling in MSPCs to the stromal and hematopoietic response to different stressors is an active area of investigation.

EXPERIMENTAL PROCEDURES

Experimental methods are briefly summarized. A detailed description is provided in Supplemental Information.

Mice

All mice were backcrossed onto a C57Bl/6 background and were maintained under standard pathogen-free conditions according to methods approved by the Washington University Animal Studies Committee. All experiments were done using 3-week-old mice unless stated otherwise. An equal number of male and female mice were used.

Cell Sorting

Hindlimbs from E16.5 mice were homogenized and digested with collagenase. TdTomato^{+} CXC12-GFP-bright, CD45^{-} CDllb^{-}, Gr1^{-}, B220^{-} cells were sorted using a Sony iCyt Synergy SY3200 cell sorter.

Micro-CT and Osmium Staining

Hindlimbs were incubated overnight at 4°C in 10% neutral buffered formalin and then embedded in 2% agarose. For osmium staining, hindlimbs were fixed with 10% neutral-buffer formalin, decalcified in 14% EDTA (pH 7.4), for 2 weeks, and then incubated in 1% osmium tetroxide for 48 h at room temperature. Processed tissues were scanned at 10-μm voxel resolution using a Scanco μCT 40.

Immunostaining of Bone Sections

Mouse hindlimbs were fixed in 4% paraformaldehyde, decalcified, incubated in 30% sucrose, and then embedded in optimal cutting temperature compound. Twelve-micron tissue sections were incubated with the Avidin/Biotin Blocking Kit (Vector Laboratories) and then incubated overnight with the indicated primary

(C and D) Representative images of E14.5 (C) or E16.5 (D) hindlimb sections from an *Osx-Cre Ai9* (i) or *Osx-Cre Tgfbr2*$^{fl/fl}$ *Ai9* (ii) mouse stained for perilipin (green), CD31 to highlight the vasculature (white), and DAPI (blue).
(E and F) Microarray was performed on *Osx-Cre*-targeted (tdTomato^{+}) cells from E16.5 hindlimbs (n = 4–6). Gene set enrichment analysis identified increased adipogenesis in cells from *Osx-Cre Tgfbr2*$^{fl/fl}$ *Ai9* mice (E). Heatmap of selected adipocyte marker genes (F).

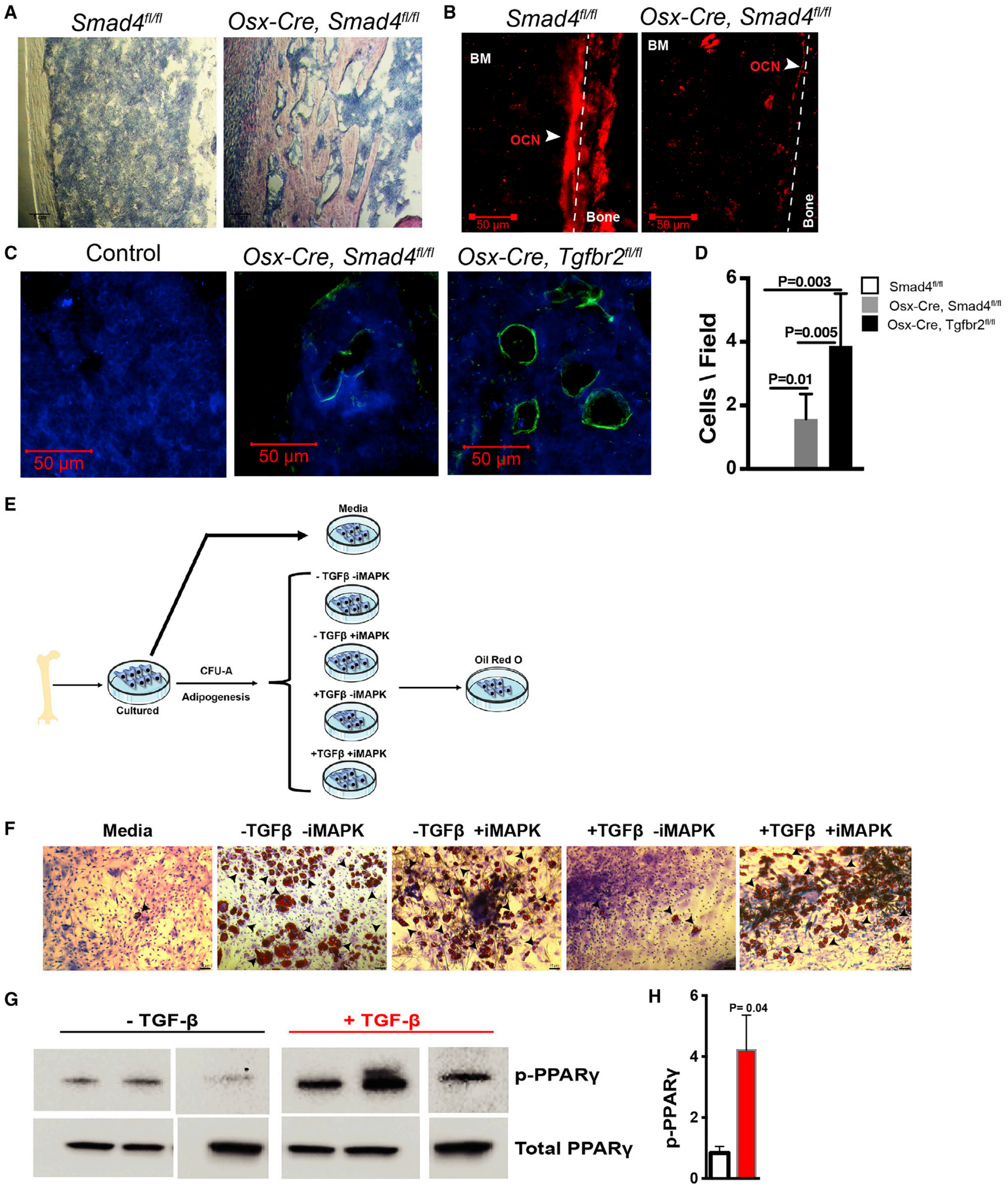

Figure 7. TGF-β Regulates Adipogenesis through Canonical and Noncanonical Pathways

(A) Representative images of H&E-stained femur sections. Original magnification 10×.

(B) Representative images of osteocalcin (red)-stained femur sections.

(C) Representative images of perilipin (green)-stained femur sections. DAPI (blue). Original magnification 40×.

(legend continued on next page)

antibodies overnight at 4°C. Images were acquired using an LSM 700 confocal microscope (Carl Zeiss Microscopy) and processed using Volocity software (PerkinElmer).

Mesenchymal Stromal Cell Culture

Hindlimbs bones from mice at E16.5 or P1–4 were mechanically disrupted and cultured overnight. Nonadherent cells were removed after 24 h. To induce adipogenesis, dexamethasone (100 nM), insulin (5 μg/mL), and indomethacin (50 mM) were added to the culture media for 5 days. Where indicated, recombinant murine TGF-β (20 ng/mL) and/or a combination of two MAPK inhibitors, U0126 (20 μM) and PD98059 (20 μM), were included in the cultures 24 h before inducing adipogenesis.

Statistical Analysis

Significance was determined using PRISM software (GraphPad), except in the case of the RNA expression profiling data, which was analyzed using the Affymetrix Transcriptome Analysis Console. For single-parameter analysis, unpaired t tests were used to assess statistical significance. For multiple parameter data, statistical significance was calculated using one-way or two-way ANOVA. The number of replicates (n) refers to individual mice, unless otherwise indicated. p values <0.05 were considered significant.

ACCESSION NUMBERS

The original microarray data from this study are available at the NCBI GEO (http://www.ncbi.nlm.nih.gov/geo/) under accession number GEO: GSE131219.

SUPPLEMENTAL INFORMATION

Supplemental Information can be found online at https://doi.org/10.1016/j.stemcr.2019.05.017.

AUTHOR CONTRIBUTIONS

G.A.-E. and D.C.L. conceived of this study. G.A.-E. performed all main experiments and analyzed the data. J.Z. and B.A. assisted with the immunohistochemistry staining. J.K. assisted with microarray analysis. C.S.C. assisted with micro-CT analysis. G.A.-E. and D.C.L. wrote the paper.

ACKNOWLEDGMENTS

This study was supported by the NIH, National Heart, Lung, and Blood Institute grant RO1HL131655 (to D.C.L.), by the NIH, National Cancer Institute grant P50CA171963, and an American Society of Hematology Scholar Award (to G.A.-E.), and by the American Society of Hematology and Edward P. Evans Foundation (to H.C.).

Received: August 29, 2018
Revised: May 15, 2019
Accepted: May 16, 2019
Published: June 13, 2019

REFERENCES

Adams, M., Reginato, M.J., Shao, D., Lazar, M.A., and Chatterjee, V.K. (1997). Transcriptional activation by peroxisome proliferator-activated receptor gamma is inhibited by phosphorylation at a consensus mitogen-activated protein kinase site. J. Biol. Chem. *272*, 5128–5132.

Alliston, T., Choy, L., Ducy, P., Karsenty, G., and Derynck, R. (2001). TGF-β-induced repression of CBFA1 by Smad3 decreases cbfa1 and osteocalcin expression and inhibits osteoblast differentiation. EMBO J. *20*, 2254–2272.

Bonde, M., Qvist, P., Fledelius, C., Riis, B.J., and Christiansen, C. (1995). Applications of an enzyme immunoassay for a new marker of bone resorption (CrossLaps): follow-up on hormone replacement therapy and osteoporosis risk assessment. J. Clin. Endocrinol. Metab. *80*, 864–868.

Bouxsein, M.L., Boyd, S.K., Christiansen, B.A., Guldberg, R.E., Jepsen, K.J., and Muller, R. (2010). Guidelines for assessment of bone microstructure in rodents using micro-computed tomography. J. Bone Miner. Res. *25*, 1468–1486.

Brenet, F., Kermani, P., Spektor, R., Rafii, S., and Scandura, J.M. (2013). TGFβ restores hematopoietic homeostasis after myelosuppressive chemotherapy. J. Exp. Med. *210*, 623–639.

Calvi, L.M., and Link, D.C. (2015). The hematopoietic stem cell niche in homeostasis and disease. Blood *126*, 2443–2451.

Camp, H.S., and Tafuri, S.R. (1997). Regulation of peroxisome proliferator-activated receptor gamma activity by mitogen-activated protein kinase. J. Biol. Chem. *272*, 10811–10816.

Choy, L., and Derynck, R. (2003). Transforming growth factor-β inhibits adipocyte differentiation by Smad3 interacting with CCAAT/enhancer-binding protein (C/EBP) and repressing C/EBP transactivation function. J. Biol. Chem. *278*, 9609–9619.

Ding, L., and Morrison, S.J. (2013). Haematopoietic stem cells and early lymphoid progenitors occupy distinct bone marrow niches. Nature *495*, 231–235.

Ding, L., Saunders, T.L., Enikolopov, G., and Morrison, S.J. (2012). Endothelial and perivascular cells maintain haematopoietic stem cells. Nature *481*, 457–462.

Greenbaum, A., Hsu, Y.-M.S., Day, R.B., Schuettpelz, L.G., Christopher, M.J., Borgerding, J.N., Nagasawa, T., and Link, D.C. (2013). CXCL12 in early mesenchymal progenitors is required for haematopoietic stem-cell maintenance. Nature *495*, 227–230.

(D) Shown is the number of perilipin+ cells per high-powered field (n = 4).
(E) Experimental schema. CFU-A represents culture conditions that favor adipocyte development. iMAPK, MAPK inhibitor.
(F) Representative images of wild-type mesenchymal stromal cell cultures stained with oil red (purple/red staining) to identify adipocytes (black arrowheads). "Media" refers to cultures not induced to adipocyte differentiation.
(G) Wild-type mesenchymal stromal cells were stimulated with TGF-β overnight and cell lysates immunoblotted for total and phosphorylated PPARγ. Each lane represents an independent culture; all samples were run on the same gel.
(H) Densitometry data for phosphorylated PPARγ normalized to total PPARγ (n = 3). Data represent the means ± SEM.

Han, J., Hajjar, D.P., Tauras, J.M., Feng, J., Gotto, A.M., Jr., and Nicholson, A.C. (2000). Transforming growth factor-beta1 (TGF-beta1) and TGF-beta2 decrease expression of CD36, the type B scavenger receptor, through mitogen-activated protein kinase phosphorylation of peroxisome proliferator-activated receptor-gamma. J. Biol. Chem. *275*, 1241–1246.

Ignotz, R.A., and Massague, J. (1985). Type beta transforming growth factor controls the adipogenic differentiation of 3T3 fibroblasts. Proc. Natl. Acad. Sci. U S A *82*, 8530–8534.

Justesen, J., Stenderup, K., Ebbesen, E.N., Mosekilde, L., Steiniche, T., and Kassem, M. (2001). Adipocyte tissue volume in bone marrow is increased with aging and in patients with osteoporosis. Biogerontology *2*, 165–171.

Kunisaki, Y., Bruns, I., Scheiermann, C., Ahmed, J., Pinho, S., Zhang, D., Mizoguchi, T., Wei, Q., Lucas, D., Ito, K., et al. (2013). Arteriolar niches maintain haematopoietic stem cell quiescence. Nature *502*, 637–643.

Logan, M., Martin, J.F., Nagy, A., Lobe, C., Olson, E.N., and Tabin, C.J. (2002). Expression of Cre Recombinase in the developing mouse limb bud driven by a Prxl enhancer. Genesis *33*, 77–80.

Maes, C., Kobayashi, T., Selig, M.K., Torrekens, S., Roth, S.I., Mackem, S., Carmeliet, G., and Kronenberg, H.M. (2010). Osteoblast precursors, but not mature osteoblasts, move into developing and fractured bones along with invading blood vessels. Dev. Cell *19*, 329–344.

Massagué, J. (2012). TGFβ signalling in context. Nat. Rev. Mol. Cell Biol. *13*, 616–630.

Mendez-Ferrer, S., Michurina, T.V., Ferraro, F., Mazloom, A.R., Macarthur, B.D., Lira, S.A., Scadden, D.T., Ma'ayan, A., Enikolopov, G.N., and Frenette, P.S. (2010). Mesenchymal and haematopoietic stem cells form a unique bone marrow niche. Nature *466*, 829–834.

Miyazawa, K., Shinozaki, M., Hara, T., Furuya, T., and Miyazono, K. (2002). Two major Smad pathways in TGF-beta superfamily signalling. Genes Cells *7*, 1191–1204.

Mizoguchi, T., Pinho, S., Ahmed, J., Kunisaki, Y., Hanoun, M., Mendelson, A., Ono, N., Kronenberg, H.M., and Frenette, P.S. (2014). Osterix marks distinct waves of primitive and definitive stromal progenitors during bone marrow development. Dev. Cell *29*, 340–349.

Naveiras, O., Nardi, V., Wenzel, P.L., Hauschka, P.V., Fahey, F., and Daley, G.Q. (2009). Bone-marrow adipocytes as negative regulators of the haematopoietic microenvironment. Nature *460*, 259–263.

Omatsu, Y., Seike, M., Sugiyama, T., Kume, T., and Nagasawa, T. (2014). Foxc1 is a critical regulator of haematopoietic stem/progenitor cell niche formation. Nature *508*, 536–540.

Qiu, T., Wu, X., Zhang, F., Clemens, T.L., Wan, M., and Cao, X. (2010). TGF-beta type II receptor phosphorylates PTH receptor to integrate bone remodelling signalling. Nat. Cell Biol. *12*, 224–234.

Scheller, E.L., Troiano, N., Vanhoutan, J.N., Bouxsein, M.A., Fretz, J.A., Xi, Y., Nelson, T., Katz, G., Berry, R., Church, C.D., et al. (2014). Use of osmium tetroxide staining with microcomputerized tomography to visualize and quantify bone marrow adipose tissue in vivo. Methods Enzymol. *537*, 123–139.

Seike, M., Omatsu, Y., Watanabe, H., Kondoh, G., and Nagasawa, T. (2018). Stem cell niche-specific Ebf3 maintains the bone marrow cavity. Genes Dev. *32*, 359–372.

Seo, H.-S., and Serra, R. (2007). Deletion of Tgfbr2 in Prx1-cre expressing mesenchyme results in defects in development of the long bones and joints. Dev. Biol. *310*, 304–316.

Sparks, R.L., Allen, B.J., and Strauss, E.E. (1992). TGF-beta blocks early but not late differentiation-specific gene expression and morphologic differentiation of 3T3 T proadipocytes. J. Cell Physiol. *150*, 568–577.

Sugiyama, T., Kohara, H., Noda, M., and Nagasawa, T. (2006). Maintenance of the hematopoietic stem cell pool by CXCL12-CXCR4 chemokine signaling in bone marrow stromal cell niches. Immunity *25*, 977–988.

Tang, Y., Wu, X., Lei, W., Pang, L., Wan, C., Shi, Z., Zhao, L., Nagy, T.R., Peng, X., Hu, J., et al. (2009). TGF-β1-induced migration of bone mesenchymal stem cells couples bone resorption and formation. Nat. Med. *15*, 757–765.

Wallace, S.E., and Wilcox, W.R. (1993). Camurati-Engelmann disease. In GeneReviews [Internet], M.P. Adam, H.H. Ardinger, R.A. Pagon, S.E. Wallace, L.J.H. Bean, K. Stephens, and A. Amemiya, eds. (University of Washington, Seattle).

Wang, Y., Cox, M.K., Coricor, G., MacDougall, M., and Serra, R. (2013). Inactivation of Tgfbr2 in Osterix-Cre expressing dental mesenchyme disrupts molar root formation. Dev. Biol. *382*, 27–37.

Wu, M., Chen, G., and Li, Y.P. (2016). TGF-beta and BMP signaling in osteoblast, skeletal development, and bone formation, homeostasis and disease. Bone Res. *4*, 16009.

Yamazaki, S., Ema, H., Karlsson, G., Yamaguchi, T., Miyoshi, H., Shioda, S., Taketo, M.M., Karlsson, S., Iwama, A., and Nakauchi, H. (2011). Nonmyelinating Schwann cells maintain hematopoietic stem cell hibernation in the bone marrow niche. Cell *147*, 1146–1158.

Yu, V.W., Lymperi, S., Oki, T., Jones, A., Swiatek, P., Vasic, R., Ferraro, F., and Scadden, D.T. (2016). Distinctive mesenchymal-parenchymal cell pairings govern B cell differentiation in the bone marrow. Stem Cell Reports *7*, 220–235.

Zhang, J., and Link, D.C. (2016). Targeting of mesenchymal stromal cells by cre-recombinase transgenes commonly used to target osteoblast lineage cells. J. Bone Min. Res. *31*, 2001–2007.

Zhang, Y.E. (2009). Non-Smad pathways in TGF-beta signaling. Cell Res. *19*, 128–139.

Zhao, M., Perry, J.M., Marshall, H., Venkatraman, A., Qian, P., He, X.C., Ahamed, J., and Li, L. (2014). Megakaryocytes maintain homeostatic quiescence and promote post-injury regeneration of hematopoietic stem cells. Nat. Med. *20*, 1321–1326.

Zhou, B.O., Yu, H., Yue, R., Zhao, Z., Rios, J.J., Naveiras, O., and Morrison, S.J. (2017). Bone marrow adipocytes promote the regeneration of stem cells and haematopoiesis by secreting SCF. Nat. Cell Biol. *19*, 891–903.

Zhou, B.O., Yue, R., Malea, M.M., Peyer, J.G., and Morrison, S.J. (2014). Leptin-receptor-expressing mesenchymal stromal cells represent the main source of bone formed by adult bone marrow. Cell Stem Cell *15*, 154–168.

Stem Cell Reports, Volume *13*

Supplemental Information

TGF-β Signaling Plays an Essential Role in the Lineage Specification of Mesenchymal Stem/Progenitor Cells in Fetal Bone Marrow

Grazia Abou-Ezzi, Teerawit Supakorndej, Jingzhu Zhang, Bryan Anthony, Joseph Krambs, Hamza Celik, Darja Karpova, Clarissa S. Craft, and Daniel C. Link

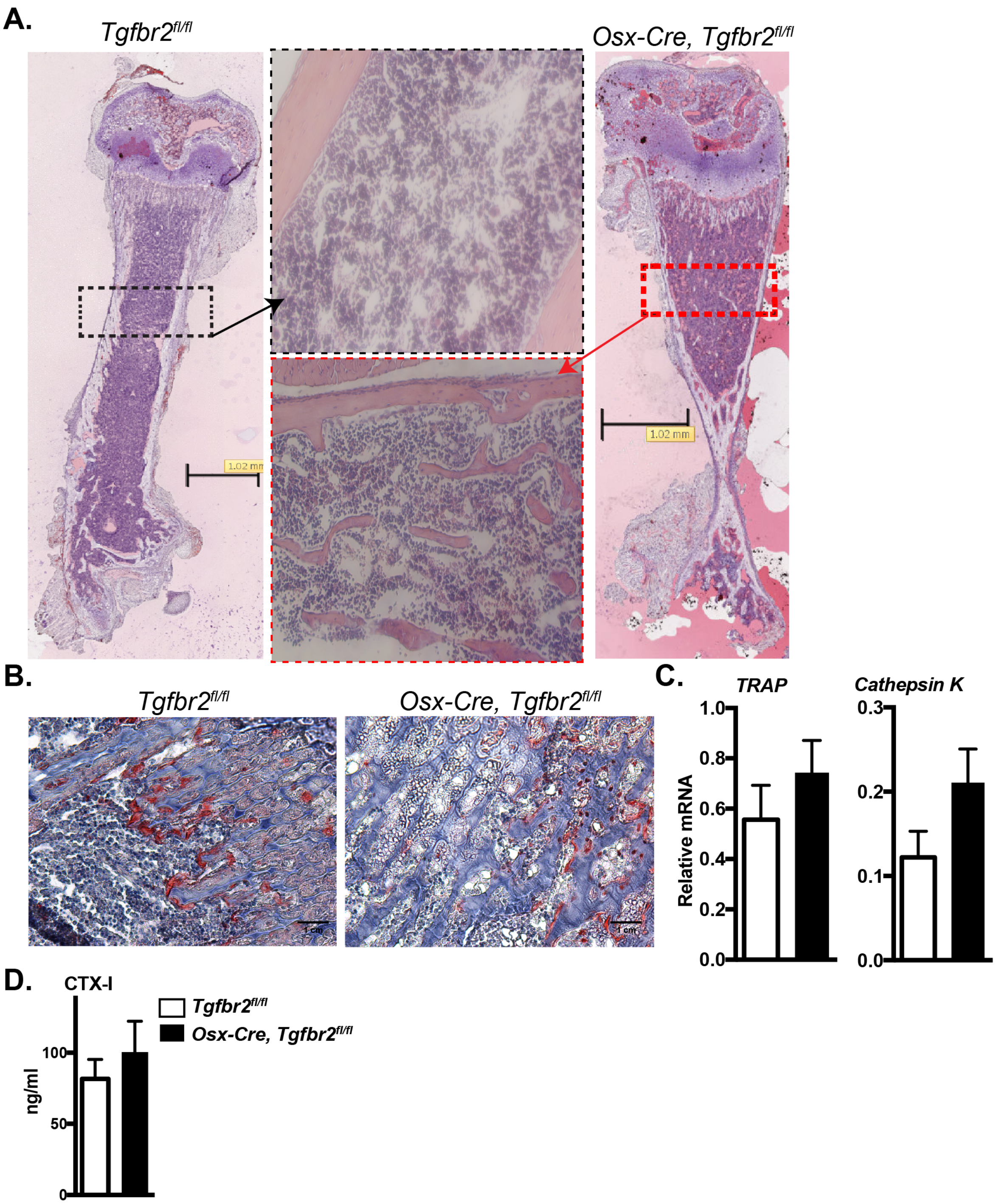
A.
Tgfbr2fl/fl
Osx-Cre, Tgfbr2fl/fl
1.02 mm
1.02 mm
B.
Tgfbr2fl/fl
Osx-Cre, Tgfbr2fl/fl
C.
TRAP
Cathepsin K
Relative mRNA
1.0
0.8
0.6
0.4
0.2
0.0
0.3
0.2
0.1
0.0
D.
CTX-I
ng/ml
100
50
0
Tgfbr2fl/fl
Osx-Cre, Tgfbr2fl/fl

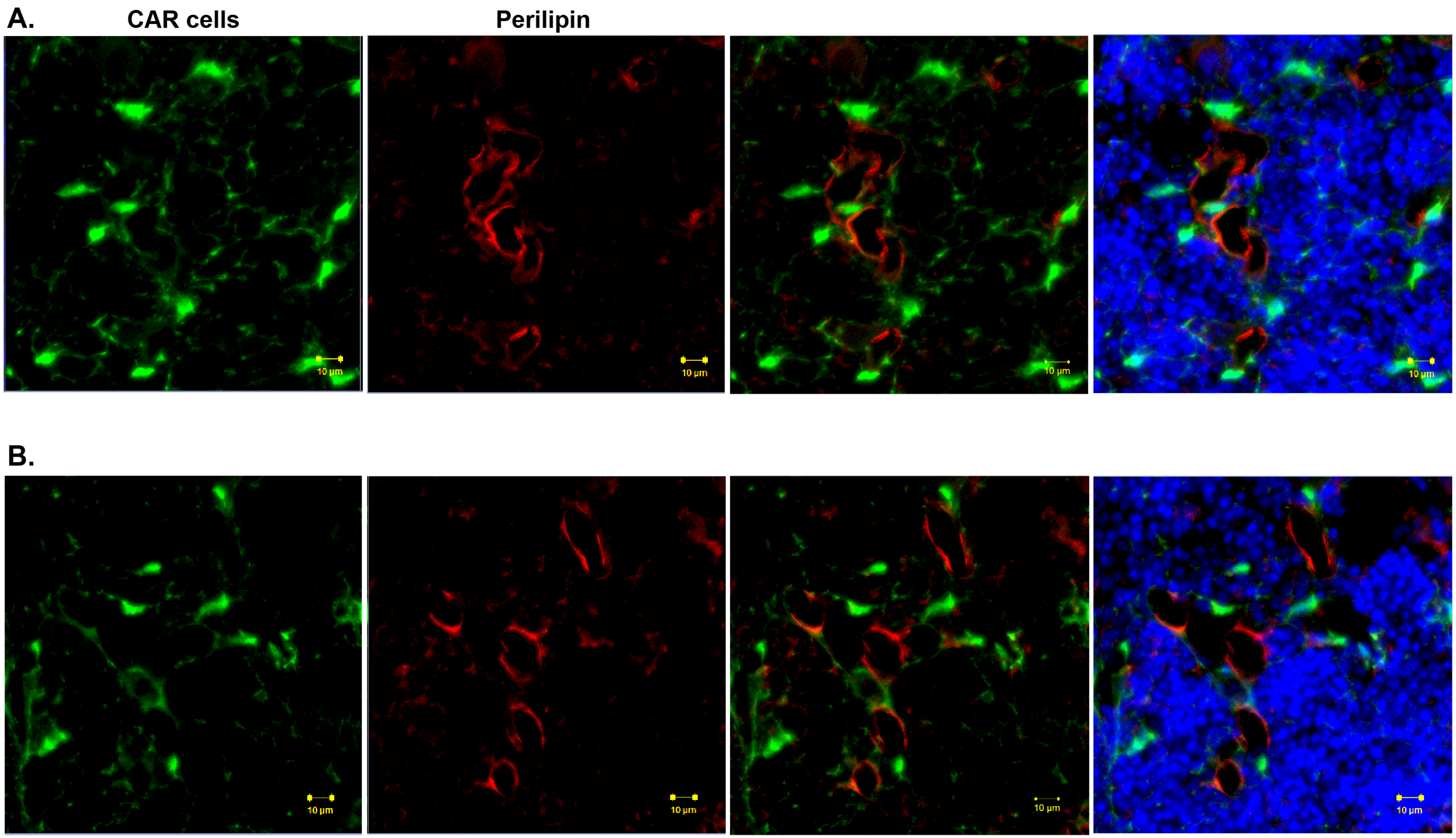
A.
CAR cells
Perilipin
10 µm
B.

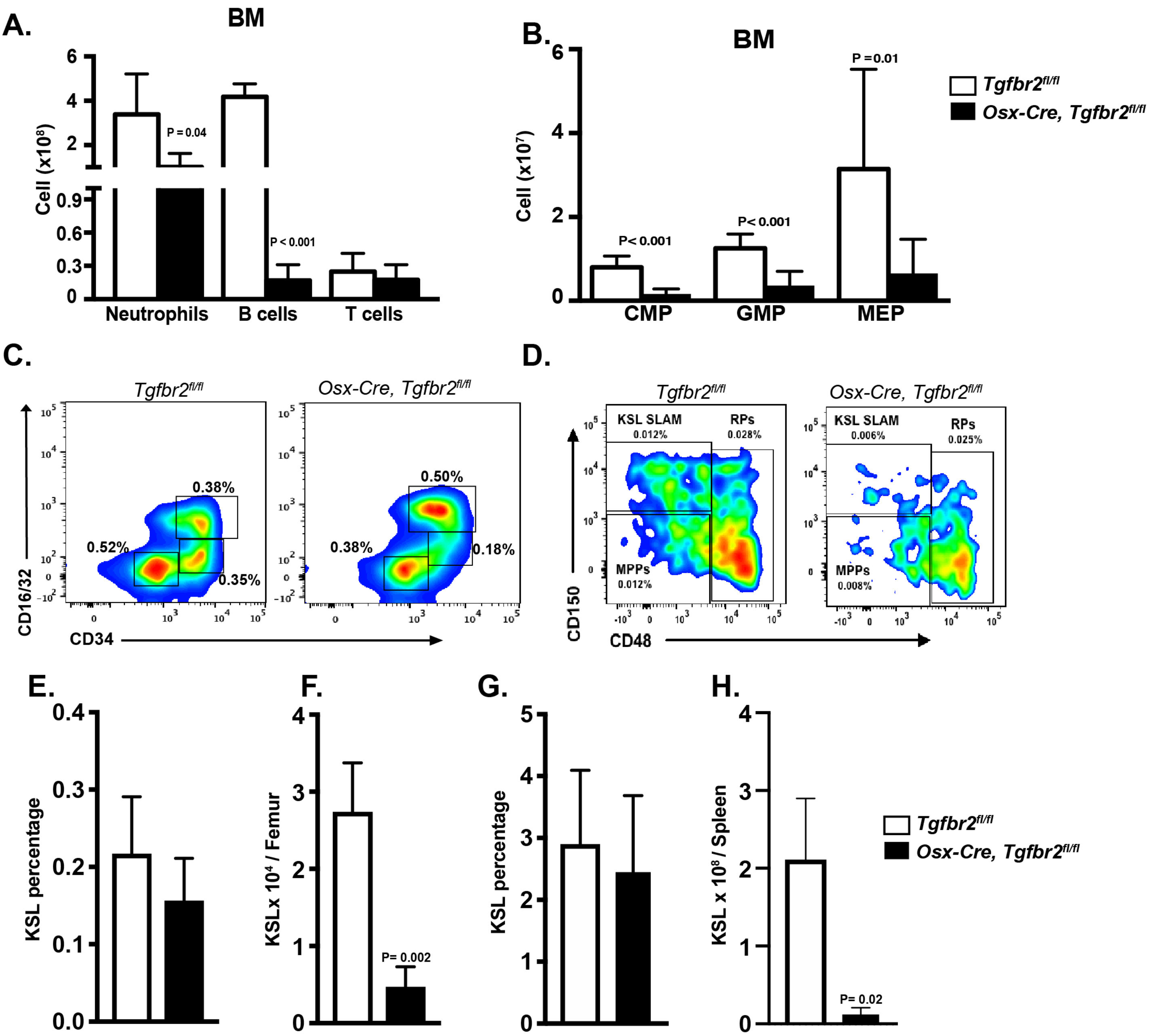
A.
BM
Cell (x10^8)
P = 0.04
P < 0.001
Neutrophils
B cells
T cells
B.
BM
Cell (x10^7)
P < 0.001
P < 0.001
P = 0.01
CMP
GMP
MEP
Tgfbr2fl/fl
Osx-Cre, Tgfbr2fl/fl
C.
Tgfbr2fl/fl
Osx-Cre, Tgfbr2fl/fl
0.38%
0.52%
0.35%
0.50%
0.38%
0.18%
CD16/32
CD34
D.
Tgfbr2fl/fl
Osx-Cre, Tgfbr2fl/fl
KSL SLAM
0.012%
RPs
0.028%
MPPs
0.012%
KSL SLAM
0.006%
RPs
0.025%
MPPs
0.008%
CD150
CD48
E.
KSL percentage
F.
KSLx 10^4 / Femur
P= 0.002
G.
KSL percentage
H.
KSL x 10^8 / Spleen
P= 0.02
Tgfbr2fl/fl
Osx-Cre, Tgfbr2fl/fl

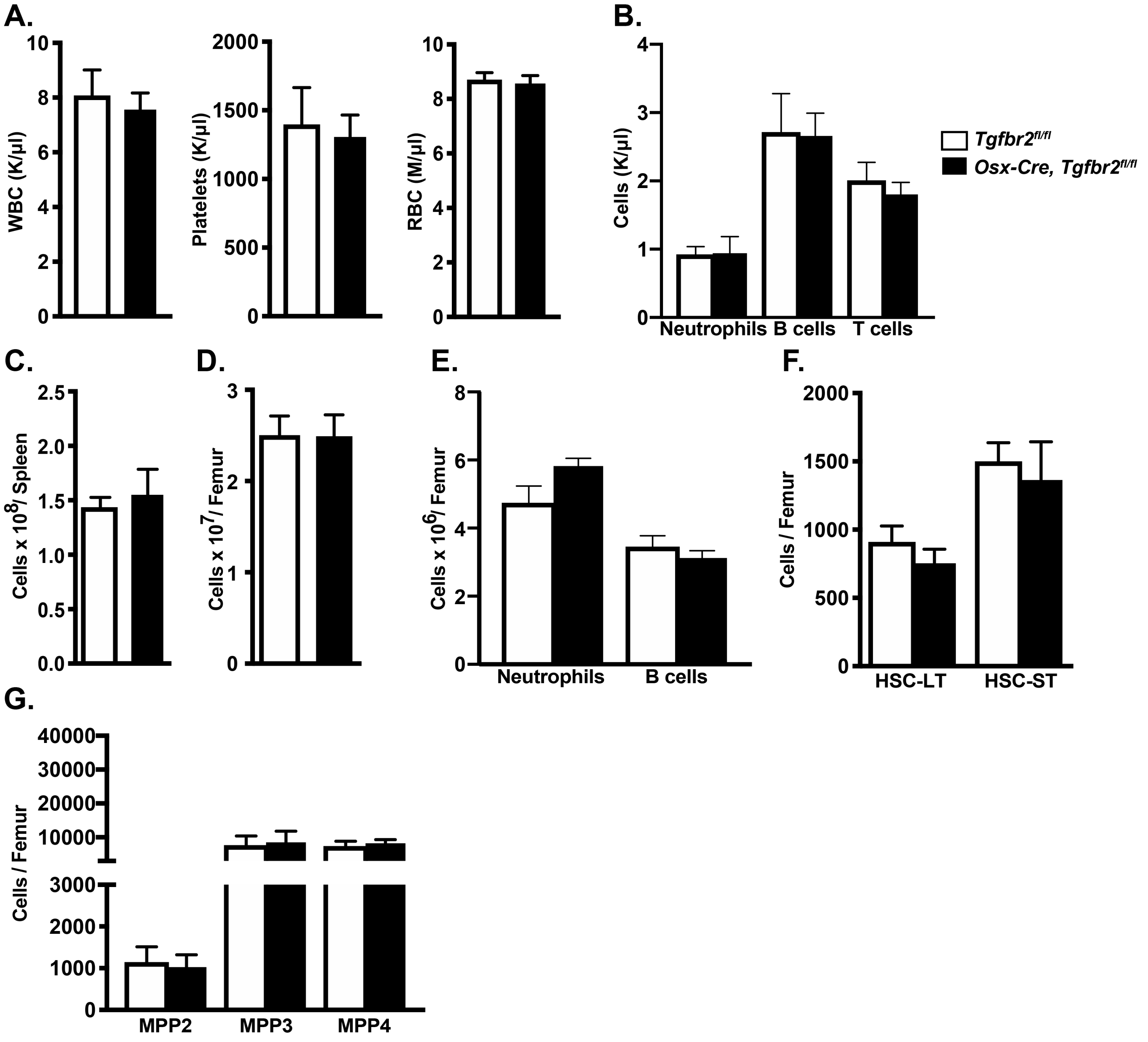

A.
WBC (K/µl)
Platelets (K/µl)
RBC (M/µl)
B.
Cells (K/µl)
Neutrophils
B cells
T cells
Tgfbr2fl/fl
Osx-Cre, Tgfbr2fl/fl
C.
Cells x 10^8/ Spleen
D.
Cells x 10^7/ Femur
E.
Cells x 10^6/ Femur
Neutrophils
B cells
F.
Cells / Femur
HSC-LT
HSC-ST
G.
Cells / Femur
MPP2
MPP3
MPP4

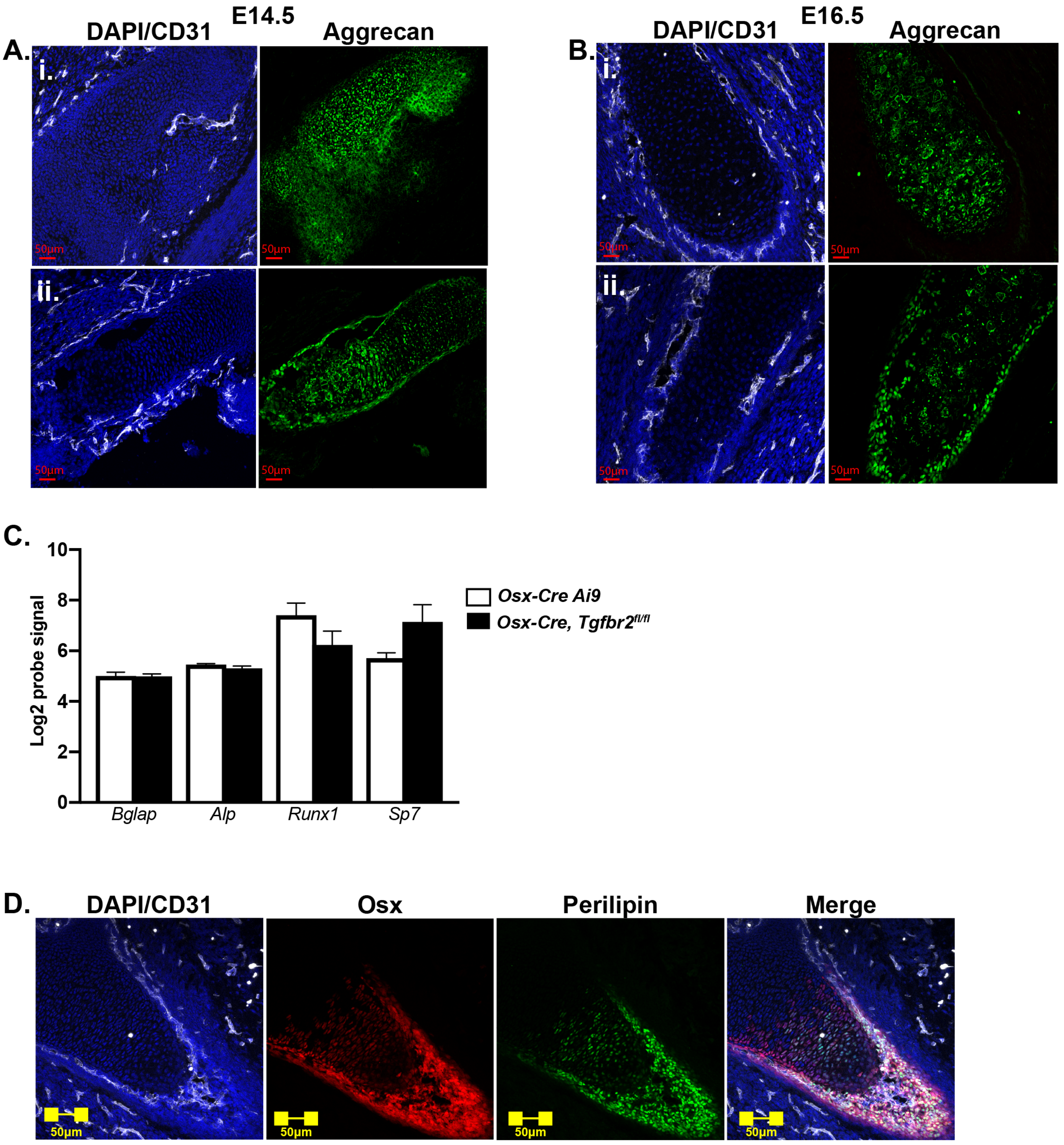
A.
E14.5
DAPI/CD31
Aggrecan
i.
ii.
50µm
B.
E16.5
DAPI/CD31
Aggrecan
i.
ii.
50µm
C.
Log2 probe signal
0
2
4
6
8
10
Bglap
Alp
Runx1
Sp7
Osx-Cre Ai9
Osx-Cre, Tgfbr2fl/fl
D.
DAPI/CD31
Osx
Perilipin
Merge
50µm

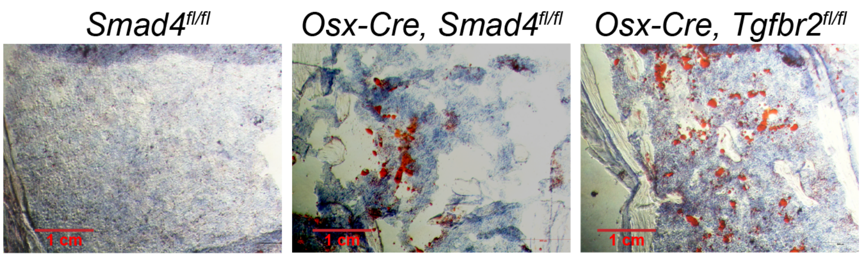
Smad4$^{fl/fl}$
Osx-Cre, Smad4$^{fl/fl}$
Osx-Cre, Tgfbr2$^{fl/fl}$
1 cm
1 cm
1 cm

Media + CFU-A | Media +CFU-A

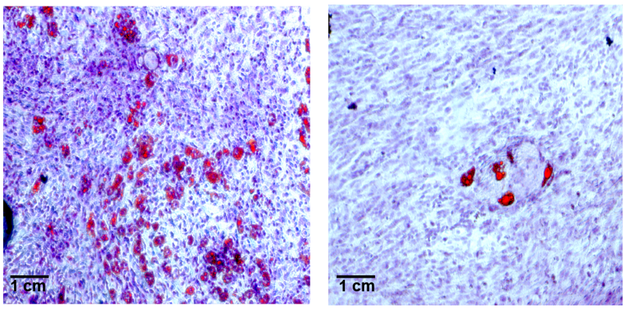

+ TGF-β

SUPPLEMENTAL FIGURE LEGENDS

Figure S1. **Related to Figure 1. Loss of TGF-β signaling in mesenchymal cells does not alter osteoclasts. A.** Representative photomicrographs of H&E stained femur sections from *Tgfbr2$^{fl/fl}$* and *Osx-CreTgfbr2$^{fl/fl}$* mice. **B.** Representative photomicrographs of femur sections stained with tartrate-resistant acidic phosphatase (TRAP, red). Original magnification 20X. **C.** RNA expression of the indicated osteoclast marker gene relative to β-actin mRNA is shown (n=5)
Osteoclasts functional genes marker of total bone marrow mRNA expression relative to β-actin. **D.** Serum C-terminal telopeptide of type I collagen (CTX-1) (n = 11-12). Data represent the mean ± SEM.

Figure S2. Related to Figure 3. CAR cells are distinct from perilipin^{+} adipocytes. (A, B) Representative images of femur sections from Tgfbr2$^{fl/fl}$ *Cxcl12^{gfp}* mice showing CXCL12-GFP (green), perilipin (red), and DAPI (blue).

Figure S3. **Related to Figure 4. Loss of TGF-β signaling in mesenchymal cells results in perturbed hematopoiesis. A-B.** The absolute number of the indicated cell type per pelvis and combined lower limbs is shown (n = 5). CMP, (common myeloid progenitor), GMP (granulocytes-macrophages progenitors), and MEP (myeloid-erythrocytes progenitors). **C**. Representative dot plots showing the gating strategy to identify CMP, GMP, and MEP; data are gated on c-Kit^{+}, lineage^{-}, Sca1^{-} cells. **D**. Representative dot plots showing the gating strategy to identify HSCs; data are gated on c-Kit^{+}, lineage^{-}, Sca1^{+} cells. RP, restricted progenitors, MPP, multipotent progenitors. **E-F.** Percentage and absolute number of bone marrow KSL cells. **G-H.** Percentage and absolute number of spleen KSL cells.

Figure S4. Related to Figure 5. Post-natal deletion of *Tgfbr2* in mesenchymal cells is not required for basal hematopoiesis. A. Peripheral blood counts. WBC, white blood cells; and RBC, red blood cells (n=6). **B.** Number of neutrophils, B cells, and T cells in the blood is shown (n=6). **C-D**. Spleen and bone marrow cellularity (per femur) (n=6). **E.** Number of neutrophils and B cells per femur. **F.** Number of HSC-LT (Lin^{-} kit^{+} Sca1^{+} CD48^{-} CD150^{+} Flt3^{-}) and HSC-ST (Lin^{-} kit^{+} Sca1^{+} CD48^{-} CD150^{-} Flt3^{-}) per femur. **G.** Number of MMP2 (Lin^{-} kit^{+} Sca1^{+} CD48^{+} CD150^{+} Flt3^{-}), MMP3 (Lin^{-} Ckit^{+} Sca1^{+} CD48^{+} CD150^{-} Flt3^{-}), and MPP4 (Lin^{-} kit^{+} Sca1^{+} CD48^{+} CD150^{-} Flt3^{+}).

Figure S5. **Related to Figure 6. TGF-β signaling in *Osx-Cre* targeted mesenchymal progenitors is not required for chondrocyte development. A-B.** Representative images E14.5 (**A**) or E16.5 (**B**) hindlimb sections from *Tgfbr2$^{fl/fl}$* (i) or *Osx-Cre, Tgfbr2$^{fl/fl}$* (ii) mice showing aggrecan (green, chondrocytes), CD31 (white, vasculature) and DAPI (blue, nuclei) staining. **C.** RNA expression profiling was performed on sorted TdTomato+ lineage- cells isolated from the hindlimb of E16.5 Osx-Cre Ai9 and Osx-*Cre, Tgfbr2$^{fl/fl}$ Ai9* mice. Shown are log2 probe signals for *Bglap, Alp, Runx1 and Sp7*. **D.** *Osterix-Cre* targeted mesenchymal cells express perilipin. Representative images of E16.5 fetal hindlimb sections from Osx-*Cre, Tgfbr2$^{fl/fl}$ Ai9* mice showing tdTomato (red, *Osx-Cre* targeted cells), CD31 (white, vasculature), perilipin (green, adipocytes cells) and DAPI (blue, nuclei) staining. Original magnitude 20X.

Figure S6. **Related to figure 7. Loss of *Smad4* in *Osx-Cre* targeted mesenchymal cells results in altered adipogenesis.** Representative images of femur sections showing oil red staining. Original magnification, 20X.

Figure S7. **Related to figure 7. TGF-β suppresses adipogenesis of cultured fetal mesenchymal progenitors.** Mesenchymal progenitors were isolated from wildtype E16.5 hindlimbs and cultured for 7 days in the (media) presence of dexamethasone, insulin and indomethacin to induce adipocyte differentiation (CFU-A). Where indicated, TGF-β was included in the culture. Shown are representative images of cultures stained for oil red to show adipocytes.

Table S1. Related to figure 6. Differentially expressed genes in *Osx-Cre* targeted (tdTomato+) cells from E16.5 hindlimbs.

Experimental Procedure

Contact for Reagents and Resource Sharing
Further information and requests for resources and reagents should be directed to and will be fulfilled by the Lead Contact, Daniel C. Link (danielclink@wustl.edu).

Experimental Model and Subject Details

Mice and Animal Housing

Osx1-GFP::Cre (Rodda and McMahon (2006), Ai9 (Madisen, Zwingman et al. 2010), *TβRII*$^{fl/fl}$ (Leveen 2002), *Dmp1-cre* (Lu, Xie et al. 2007), and *Smad4*$^{fl/fl}$ (*Smad4*$^{tm2.1Cxd}$/J) mice were obtained from The Jackson Laboratory. *Cxcl12*^{gfp} mice (Nagasawa, Hirota et al. 1996) were a gift from Dr.Takashi Nagasawa (Kyoto University, Japan). Mice were crossed in-house to generate *Osx-Cre Tgfbr2*$^{fl/fl}$*, Osx-Cre Tgfbr2*$^{fl/fl/Ai9}$*, Osx-Cre Tgfbr2*$^{fl/fl}$*Cxcl12*^{gfp}*, Osx-Cre*^{Ai9}*, Dmp-1-Cre Tgfbr2*$^{fl/fl}$*, and Osx-Cre Smad4*$^{fl/fl}$ Ai9 mice on a C57Bl/6 background. To suppress the *Osx-Cre* transgene, mice were maintained on doxycycline chow (200 mg/serving). All experiments were done using 3 weeks old mice unless stated otherwise. An equal number of male and female mice were used. Mice were maintained under standard pathogen-free conditions according to methods approved by the Washington University Animal Studies Committee.

Flow cytometry

Peripheral blood, bone marrow, and spleen mononuclear cell preparations were lysed in Tris-buffered ammonium chloride (pH 7.2) buffer for 5 minutes at 4°C. Cells were then incubated with the indicated antibody at 4°C for 30 minutes in phosphate buffered saline (PBS) containing 1mM ethylenediaminetetraacetic acid (EDTA) and 0.2% (weight/volume) bovine serum albumin (BSA). The HSPC panel included Pe-Cy7-conjugated CD117 (2B8); BV711-conjugated Ly-6A/E; BV605-conjugated CD150 (TC15-12F12.2) BV421-conjugated CD48 (HM48-1); APC-conjugated CD16/32 (2.4G2); FITC-conjugated CD34 (RAM34); and PE-conjugated CD135 (A2F10.1) and the following APC-Cy7-conjugated antibodies: CD3e (145-2C11), B220 (RA3-6B2), Gr1 (RB6-8C5), Ter119 (TER-119), and CD11b (M1/70). The CAR cell panel included the following BV421-conjugated antibodies against CD45 (30-F11), B220, Gr1, and CD11b. Cells were acquired using a FACSAria III flow cytometer (BD biosciences, San Jose, CA, USA) and analyzed using FlowJo software.

Cell sorting

Hindlimbs and forelimbs from embryonic day E16.5 were homogenized in PBS using a mortar and pestle. The cell suspension was then incubated in PBS containing 1.7 mg/ml collagenase type 1 (#17100017, ThermoFisher, Waltham, MA, USA), 1.7 mg/ml collagenase type 2 (#LS004174, Worthington, Lakewood, NJ, USA), and 1.7 mg/ml collagenase type 4 (#LS004188, Worthington) at 37°C while shaking for 10 minutes. The resulting cell suspension was filtered through CellTrics 50µm filters (Sysmex, Goerlitz, Germany) to remove cell clumps and then incubated at 4°C for 30 minutes in PBS containing 1mM EDTA and 0.2% BSA with the following panel of FITC-conjugated lineage antibodies: CD45, Gr1, CD11b, and B220. Tdtomato^{+} lineage^{-} cells were sorted using a Sony iCyt Synergy SY3200 (Synergy)" cell sorter (Sony, San Jose, CA, USA). CXCL12-GFP-bright CD45^{-} lineage^{-} cells were sorted from *Osx-Cre Tgfbr2*$^{fl/fl}$*Cxcl12*^{gfp} *or Tgfbr2*$^{fl/fl}$*Cxcl12*^{gfp} mice using a similar strategy.

Micro-computed tomography and osmium staining

Hindlimbs were incubated overnight at 4°C in 10% neutral buffered formalin and then embedded in 2% agarose. Femurs were scanned at 10µm voxel resolution, using a Scanco µCT 40 (Scanco Medical, Wayne, PA) calibrated using a hydroxyapatite phantom. Measurements of both cancellous and cortical bones were made based on reported guidelines (Bouxsein, Boyd et al. 2010). For cancellous bone, 100 slices (1mm) proximal to the growth plate (GP) were contoured to exclude the cortical bone, allowing cancellous bone volume/tissue volume (BV/TV) and bone mineral density (BMD) to be determined. For cortical bone, 20 slices (200 µm) located mid-diaphysis were contoured to exclude the marrow space and analyzed to determine cortical tissue mineral density (TMD) and cortical bone thickness. A threshold of 260 for cortical bone (on a 0–1000 scale) was maintained. For cancellous bone, a threshold of 175 was used for µCT. An additional analysis of cancellous bone was performed within the cortical bone region of interest after discovering the presence of cancellous bone mid-diaphysis in the mutant, but not control bones. Like cancellous bone at the GP, femurs were contoured to exclude the cortical bone and a threshold of 175 was maintained.

For osmium staining, hindlimbs were fixed with 10% neutral-buffer formalin, washed with water and decalcified in 14% EDTA, Ph 7.4, for 2 weeks. After washing again with water, 600µl Sorensen's phosphate buffer (pH 7.4) was added and then the hindlimbs incubated in a fume hood with 1% osmium tetroxide for 48 hours at room temperature. Hindlimbs were washed three times by incubating in 1 ml of Sorensen's buffer for 3 hours at room temperature with the last wash including an overnight incubation at room temperature.

Immunostaining of bone sections

Mouse hindlimbs were fixed in PBS containing 4% paraformaldehyde, pH 7.4, for 24 hours at 4°C. Bones were then decalcified in PBS containing 14% EDTA, pH 7.4, for 7 days at 4°C. Following incubation in PBS containing 30%

sucrose for 24 hours at 4°C, bones were embedded in optimal cutting temperature compound (OCT) (Sakura Finetek, Torrance, CA, USA). The tissue blocks were cut into 12µm sections using a Leica Cryo-Jane system (Leica Biosystems, Wetzlar, Germany). For immunostaining, the slides were first incubated in 0.1M Tris-Cl pH 7.5, 150mM NaCl, and 0.1% Tween 20 (TNT) buffer containing 10% donkey serum for 1 hour at room temperature. Sections were then incubated for 15 minutes at room temperature using the Avidin/Biotin Blocking Kit (SP-2001; Vector Laboratories, Burlingame, CA, USA). Sections were incubated with the primary antibody overnight at 4°C and, where applicable, then incubated with the secondary antibody at a 1 to 100 dilution for 1 hour at room temperature. The following antibodies were used: rabbit anti-perilipin at a 1:800 dilution (MilliporeSigma), anti-PECAM-1 at a 1:100 dilution (clone 2H8, MilliporeSigma), rabbit anti-aggrecan at a 1:100 dilution (AB1031, MilliporeSigma), and rabbit anti-osteocalcin at a 1:50 dilution (ab10911, ABCAM, Cambridge, United Kingdom). Finally, slides were mounted with ProLong Gold antifade reagent with DAPI (Life Technologies, Inc., Grand Island, NY, USA). Images were acquired using an LSM 700 confocal microscope (Carl Zeiss Microscopy, Peabody, MA, USA) and processed using Volocity software (PerkinElmer, Waltham, MA, USA).

Hematoxylin and eosin (H&E) staining of bone sections was performed using the Hematoxylin and Eosin Stain Kit (Vector Laboratories Burlingame, Ca. USA, Cat# H-3502). Oil red staining was performed using the Sigma Oil Red O kit per manufacturer's recommendations (MilliporeSigma). Sections were mounted with Organo/Limonene Mount™ (MilliporeSigma), and images were acquired using an Hamamatsu Nanozoomer (Hamamatsu Photonics, Hamamatsu City, Japan).

Quantitative reverse-transcription PCR

Total bone marrow RNA was obtained by flushing femurs with 1 ml of Trizol (Invitrogen). RNA was prepared according to the manufacturer's specification. One-step quantitative reverse-transcription PCR was performed using the TaqMan Universal PCR Master Mix (Applied Biosystems) using no template and no reverse-transcription controls. Data was collected on a 7300 Real-Time PCR System (Applied Biosystems). Oligonucleotides are provided in the Key Resource.

RNA expression profiling

RNA was purified from sorted mesenchymal cells using the NucleoSpin® RNA XS kit per manufacturer's recommendations (MACHEREY-NAGEL, Düren, Germany). Libraries were prepared using the Affymetrix WT Pico kit per manufacturer's recommendations (Affymetrix, Waltham, MA, USA) and then hybridized onto Affymetrix Clariom S arrays. The arrays were placed in a GeneChip Hybridization Oven 640 for 18 hours at 45°C, and then they were washed and stained in an Affymetrix Fluidics Station 450. The arrays were scanned using the Affymetrix GeneChip 7G 3000 Scanner. Data were analyzed on an Affymetrix GeneChip Command Console to generate CEL files. Affymetrix Transcriptome Analysis Console (TAC) software was used to treat the CEL files through Robust Multichip Analysis (RMA) algorithm, including probe-set signal integration, background correction and quantile normalization. TAC software also was used to identify differentially expressed genes. In this study, differentially expressed genes were defined by the following criteria: 1) false discovery rate (FDR) of $\leq$ 7.5%; 2) fold-change of $\geq$ 2; and 3) a minimum probe signal of 50 in the upregulated group. To identify the significantly enrichment of gene ontology (GO) terms and functional pathways, DAVID (http://david.abcc.ncifcrf.gov/tools.jsp), Kyoto Encyclopedia of Genes and Genomes (KEGG, http://www.genome.jp/kegg/), and pre-ranked Gene Set Enrichment Analysis (GSEA, http://software.broadinstitute.org/gsea/index.jsp) were used.

Mesenchymal stromal cell culture

Mice were sacrificed on embryonic day 16.5 (E16.5) or 1-4 days after birth, and hindlimb bones were harvested. Bones were mechanically disrupted in complete Dulbecco's modified eagle medium (DMEM) plus 20% fetal bovine serum (FBS) and penicillin-streptomycin using a mortar and pestle. The resulting cell suspension was cultured overnight at 37°C with 5% CO_2, and the following day non-adherent cells removed by gentle aspiration. Cells were cultured until reaching approximately 40% confluence (generally, 5-6 days). To induce adipogenesis, dexamethasone (100nM), insulin (5 µg/ml), and indomethacin (50 mM) were added to the culture media for 5 days. Where indicated, recombinant murine TGF-β (20ng/ml) and/or a combination of two MAPK inhibitors, U0126 (20 µM) and PD98059 (20 µM), were included in the cultures 24 hours prior to inducing adipogenesis.

Western Blot

Total cellular protein was isolated from culture mesenchymal cells using RIPA lysis buffer (#20-188, MilliporeSigma) inhibiting for protease and phosphatase (#78440, ThermoFisher). Twenty µg of protein was separated using pre-cast 4-15% gradient SDS gels (#456-1084, Bio-Rad) and transferred to nitrocellulose membranes (#IPVH00010.

Millipore). Membranes were incubated with a 1:1000 dilution of rabbit anti-PPARγ (PA3-821A, MilliporeSigma) or a 1:1000 dilution of rabbit anti-phospo (Ser82)-PPARγ) (#04-816, MilliporeSigma) in Tris buffered saline 100mM Tris HCl, 1.5M NaCl, pH 7.5 (TBS) (#P029, G-biosciences, Saint Louis, MO, USA) and 5% BSA (TBS) overnight at 4°C. Following multiple TBS washes, the membranes were incubated with a 1:5000 dilution of horseradish-peroxidase-conjugated anti-rabbit antibody (#A0545, MilliporeSigma) for 1 hour at room temperature. Following incubation with a chemiluminescence horse radish peroxidase substrate (Millipore, #WBKLS0100), the membranes were imaged using the Bio-Rad ChemiDoc XRS+ (BIO-RAD, Hercules, CA, USA.).

Quantification and Statistical Analysis

Significance was determined using PRISM software (GraphPad), except in the case of the RNA expression profiling data, which was analyzed using the Affymetrix Transcriptome Analysis Console. For single parameter analysis, unpaired t-test were used to assess statistical significance. For multiple parameter data, statistical significance was calculated using one-way or two-way analysis of variance (ANOVA). P values less than 0.05 were considered significant.

Supplemental References

Rodda, S. J. and A. P. McMahon (2006). "Distinct roles for Hedgehog and canonical Wnt signaling in specification, differentiation and maintenance of osteoblast progenitors." Development **133**(16): 3231-3244.

Madisen, L., T. A. Zwingman, S. M. Sunkin, S. W. Oh, H. A. Zariwala, H. Gu, L. L. Ng, R. D. Palmiter, M. J. Hawrylycz, A. R. Jones, E. S. Lein and H. Zeng (2010). "A robust and high-throughput Cre reporting and characterization system for the whole mouse brain." Nat Neurosci **13**(1): 133-140.

Leveen, P. (2002). "Induced disruption of the transforming growth factor beta type II receptor gene in mice causes a lethal inflammatory disorder that is transplantable." Blood **100**(2): 560-568.

Lu, Y., Y. Xie, S. Zhang, V. Dusevich, L. F. Bonewald and J. Q. Feng (2007). "DMP1-targeted Cre expression in odontoblasts and osteocytes." J Dent Res **86**(4): 320-325.

Nagasawa, T., S. Hirota, K. Tachibana, N. Takakura, S. Nishikawa, Y. Kitamura, N. Yoshida, H. Kikutani and T. Kishimoto (1996). "Defects of B-cell lymphopoiesis and bone-marrow myelopoiesis in mice lacking the CXC chemokine PBSF/SDF-1." Nature **382**(6592): 635-638.
